# A DFT and Matrix–Isolation IR/UV-Visible Study of High-Coordinated Lanthanide-CO Complexes

**DOI:** 10.3390/molecules28135043

**Published:** 2023-06-28

**Authors:** Attila Kovács, Werner Klotzbücher

**Affiliations:** 1 European Commission, Joint Research Centre (JRC), Karlsruhe, Germany; 2 Former Max Planck Institute for Radiation Chemistry, 45470 Mülheim a.d. Ruhr, Germany; werner.klotzbuecher@cec.mpg.de

**Keywords:** lanthanides, carbonyl complexes, matrix–isolation spectroscopy, FT-IR, UV-visible, DFT

## Abstract

Recent joint mass spectrometric and IR photodissociation studies have provided proof on the existence of octa-coordinated ionic lanthanide-carbonyl complexes under those extreme gaseous conditions. In contrast, in older literature concerning cryogenic studies of neutral Ln(CO)_x_ species, the highest coordination was assigned to hexa-coordinated Ln(CO)_6_ molecules. The present study aims to clarify the above controversy using matrix isolation spectroscopy and DFT calculations. In order to ensure the maximum possible coordination, the Ln(CO)_x_ complexes were synthesized in neat CO cryogenic matrices at 10 K and were investigated by infrared and UV-visible spectroscopy. The formed complexes were identified on the basis of the characteristic CO stretching frequencies of the ground-state molecules predicted by DFT calculations. Our joint experimental–theoretical analysis confirmed the preference of octa-coordinated Ln(CO)_8_ complexes in cryogenic neat CO matrices.

## 1. Introduction

Transition metal complexes are ubiquitous in today’s chemistry due to their ability to switch between different oxidation states and the possibility to fine-tune their reactivity and selectivity by external factors. Numerous reviews and books covered the progress over the years, and one recently garnered the advances in catalysis of more than 50 groups worldwide [1]. Other examples are found in the use of transition metal oxides in lithium-ion batteries or the nanomaterials of other energy-related applications [2].

As a sub-set, transition metal carbonyls have numerous applications such as industrial catalysts or precursors for complicated compounds. The study of their characteristics was vastly advanced in the 1970s with the introduction of a combination of matrix isolation trapping, spectroscopic investigation, and photochemical excitation. Within a few years, binary stable and unstable species of most of the transition metals (TM) were discovered by one of two routes: either the trapping of unstable intermediates after the photochemical reaction of a stable precursor in inert matrices or the direct formation of species of interest by the direct reaction of “naked” metal atoms with CO molecules diluted in inert gas mixtures prior to freeze-out on a matrix window. Techniques and discovered complexes have been topics in numerous articles, reviews, and books [3,4,5,6].

In general, only TM metals with an even atomic number build neutral mononuclear complexes, such as Fe(CO)_5_, Ni(CO)_4_, and their homologues. Notably, TM(CO)_1–6_ (TM = Cr, Mo, W) [7], where the Cr, Mo, and W hexacarbonyls are stable (crystalline) compounds at room temperature, have a well-characterized octahedral structure and follow the so-called 18-electron rule. However, the validity of this rule was questioned, as the adjacent V(CO)_6_ is also stable and octahedral [8,9] but only has seventeen valence electrons. More doubts arose when the generation of matrix-isolated Ti(CO)_6_ with only sixteen valence electrons was reported in 1977 [10]. The possibility of the mis-assignment of hexa-coordinated Ti(CO)_6_ was elaborated in detail by the group of Schaefer III, who stressed the importance of the 18-electron rule in 2008 by calculating the bonding of seven carbonyls to a single TM atom M(CO)_n_ (M = Ti, Zr, Hf; n = 7, 6, 5, 4), which fulfilled the 18-electron rule [11]. A comprehensive analysis of the bonding in TM carbonyl complexes was reported in Ref. [12].

By contrast, very little is known about carbonyl species of the lanthanides and actinides. For some time it was believed that they could not be synthesized. However, in 1971, the group of Weltner reported the synthesis of uranium carbonyl [13] by the matrix isolation route, and soon after the group of DeVore the detection of neodymium and ytterbium carbonyls [14] by the same technique, followed by praseodymium, europium, gadolinium, and holmium carbonyls [15]. Still, this did not raise a great deal of interest. In the 1980s only one Soviet group reported further data [16], and in the 1990s only a joint experimental/theoretical paper on Sm atom reactions with inert and reactive matrices appeared [17]. This changed around the turn of the century, when laboratories in the United States, China, and Japan started to investigate reactions of laser-ablated actinide [18] and lanthanide atoms with CO [19,20,21], culminating in the 2014 report on octa-coordinated lanthanum and cerium carbonyl [22].

Early matrix–IR spectra of f-elements complexed with CO were interpreted on the basis of experiences with the transition metals. Thus, from their first detection in the 1970s, six was considered the maximum coordination number for the binary CO complexes of f-elements. This influenced all quantum chemical calculations in the 1980s and 1990s [23,24,25,26,27]. More sophisticated experimental and theoretical studies on f-element carbonyls started in the 21st century: joint MI-IR and advanced DFT studies were performed on the reactions of CO with laser-ablated La [21,28], Gd [29], and Ce [20]. For the first time, higher coordinated species [21,29] were discussed. Monocarbonyl complexes of all rare earth elements (Sc, Y, La-Lu) were assessed by a joint MI-IR and sophisticated quantum chemical (DFT, CASPT2, CCSD(T)) analysis [30].

The formation of ionic octa-coordinated La and Ce carbonyl complexes under extreme conditions was first reported by Xie et al. [22]. Following a similar earlier procedure resulting in the observation of U(CO)_8_^+^ in the gaseous phase [31], the La(CO)_8_^+^ and Ce(CO)_8_^+^ ions were detected by mass spectroscopy and the CO stretching vibrational frequencies were measured by infrared photodissociation spectroscopy. The experimental results were supported by DFT calculations [22]. In subsequent years, octa-coordinated CO complexes of alkaline earths (Ca, Sr, Ba) [32,33,34], of heavy transition metals (Zr, Hf) [35], of rare earths (Sc, Y, La, Tm, Yb, Lu) [36,37,38], and of actinides (Th, U) [39] were observed and characterized using matrix–isolation spectroscopy [32,35] and gas-phase mass-selected infrared photodissociation spectroscopy [33,34,36,37,38,39] jointly with DFT calculations.

The formation of ionic octa-coordinated CO complexes in the gaseous phase is already well-proven. Less reliable information is available on the formation of the highest-coordinated neutral complexes, as all earlier papers assigned the potential absorption bands in the MI-IR spectra to the hexa-coordinated species [14,15,17,21,27] or left them unassigned [20,28].

Neutral complexes (without ionic and excited-state by-products) can be synthesized by the reaction of thermally evaporated Ln atoms with pure CO gas, where the pure CO surroundings enhances the formation of the high-coordinated Ln(CO)_x_ species. Performing such experiments in the present study, we focus here on the identification of the high-coordination Ln(CO)_x_ complexes on the basis of their characteristic CO stretching vibrations and UV-visible spectra. The assignments are assisted by DFT calculations.

## 2. Results and Discussion

### 2.1. Experimental Results

**Figure 1:**
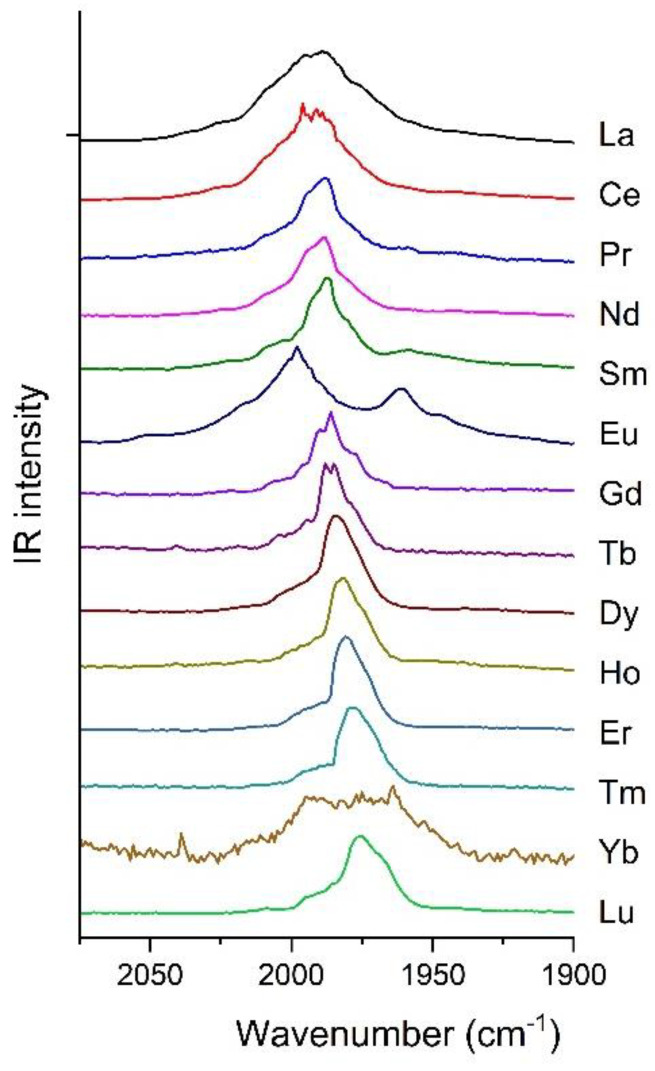
IR spectra of lanthanide atoms deposited with neat CO in the characteristic 2075–1900 cm^−1^ range.

The IR spectra of depositions of lanthanide atoms with neat CO characteristically exhibits a broad band centered between 1990 to 1975 cm^−1^ (Figure 1). In general, this feature sharpened slightly upon annealing and was stable upon photoactivation.

UV-visible spectra provided interesting additional information on the formation reaction of lanthanide carbonyls in the experiments. Most UV spectra indicated a full reaction upon deposition in neat CO. No sign of the characteristic narrow-band features of unreacted (“naked”) lanthanide atoms was visible. Some exceptions were traces of atomic features in the samarium, dysprosium, and erbium depositions, and a strong spectrum of unreacted atoms in the cases of thulium and ytterbium. In all spectra with atomic features these vanished upon brief irradiation into the strongest atomic absorption, accompanied by the growth of the much broader band associated with the molecular species.

The above observation points to a fast and preferred reactivity of most thermal Ln atoms with CO in the gaseous and semi-liquid phase immediately before trapping on the optical window.

A compilation of the absorption bands in the UV-visible spectra and the characteristic UV-visible and FT-IR spectra of all the deposited Ln atoms is given in the Supplementary Material.

### 2.2. Structures and Electronic Properties of Ln(CO)_8_ Complexes

Selected B3LYP/VTZP data on the computed most stable Ln(CO)_8_ complexes are given in Table 1. Comparing the obtained lowest-energy states from the B3LYP calculations to those of the neutral Ln atoms from the experiment [40,41] (Table S1), we could observe that only part of the Ln atomic ground states was preserved upon complexation with CO. These are those of La, Pr, Nd, Gd, Yb, and Lu. For several neutral Ln atoms (Ce, Tb, Dy, Ho, Er, Tm), the lowest probed spin multiplicity was reported for the ground state [40,41]. Unfortunately, our DFT calculations on these low-spin complexes suffered from high spin contamination, and therefore these computed results cannot be trusted. They are given only in the Supplementary Material (Table S2) for information purposes.

The complex formation changes the electron configuration of the Ln atoms. As determined by NBO analysis (Table 1), the populations of the 6s orbitals decrease considerably, although these are hardly suitable for donor–acceptor interactions. Apparently, in order to facilitate optimal Ln→CO back-donation, the Ln atoms have to be promoted from the ground states that havea 6s^2^ population to higher-energy states with more advanced 5d populations. Therefore, the natural populations of Ln indicate a decrease from 6s^2^ by 1.0–1.6 e.

At the same time, the 5d populations are around 5d^3^ for most Ln complexes, whereas in the atomic ground states it was 5d^1^ for La, Ce, Gd, and Lu or 5d^0^ for all the other Ln (see Table S1). The promotion includes a rearrangement (significant decrease) of the 4f populations in the case of Pr, Nd, Tb, Dy, Ho, Er, and Tm. Some exceptions that have roughly 5d^2^ populations in the complexes are Sm and Eu, where the 4f^6^ and 4f^7^ populations were retained, respectively. The latter two cases well-characterize the preference of the (near) half-filled 4f shell in Ln: the decrease of the 4f^6^ and 4f^7^ populations would require too much energy (not covered by the Ln→CO back-donation). From the second part of the Ln row, the 4f population of Tb (7.42 e) is decreased considerably from 4f^9^ of the sextet atomic Tb ground-state (or from the low-lying octet 4f^8^5d^1^ state; see Table S1). This feature points to the preference for the half-filled 4f shell. A related (and well-known) situation occurs in Yb, where the 4f^14^ configuration forms the ground state in both the neutral Yb atoms and in the Yb(CO)_8_ complex.

**Figure 2:**
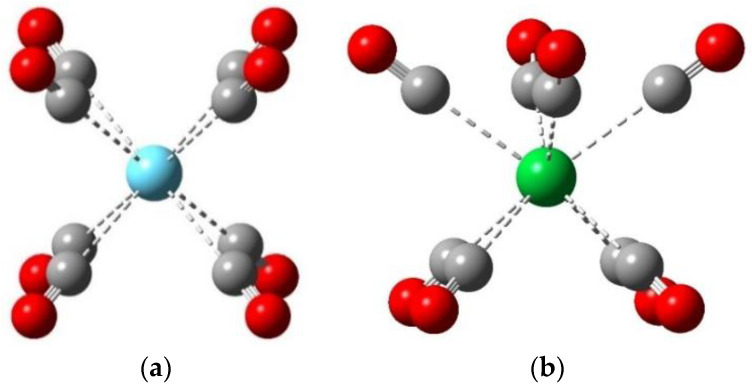
Characteristic computed structures of Ln(CO)_8_ complexes: (**a**) D_4h_ of ^2^La(CO)_8_; (**b**) D_4d_ of ^1^Yb(CO)_8_. The atomic colors are as follows: cyan, La; green, Yb; gray, C; red, O.

In most cases, the used B3LYP/VTZP level resulted in highly symmetric Ln(CO)_8_ structures (Figure 2), similarly to the recent results on the Ln(CO)_8_^−^ and Ln(CO)_8_^+^ ionic species [36,37,38]. The O_h_ symmetry was manifested for Sm and Eu, whereas it was slightly distorted to D_4h_ in the case of most other Ln. A significantly different D_4d_ arrangement was obtained for Yb (cf. Figure 2b), which was attributed to the exceptional divalent character of this metal.

In the case of Tb and Lu, the obtained structures (D_2d_ and D_2h_, respectively) were slightly distorted from the high symmetry that was mostly observed (cf. Table 1). Moreover, for Dy(CO)_8_, a markedly distorted C_s_ structure (with 0.004 Å deviation between the two different Dy-C bonds) was obtained. We note the experienced extreme SCF problems in the case of the Dy complex, which might also be due to its strong multiconfigurational character—it implies significant deficiencies of DFT to model the electronic structure of this molecule.

The well-known ‘lanthanide contraction’ feature [42] can be recognized in most Ln-C bond distances. Deviations towards longer Ln-C bonds occur in the Sm, Eu, Gd, and Yb complexes as a probable consequence of the preference for a half- or fully-filled 4f shell.

A very important insight on the geometrical properties is that significantly different Ln-C bond distances leave the C≡O bond distance intact. For example, a change of the Eu-C bond up to 0.05 Å leaves the C≡O bond distance at 1.135 Å, and the vibrational frequencies are only marginally different (cf. Table S2). This confirms the very strong character of the C≡O bond. More evidence for this character is that, compared with Ln(N_2_)_x_ complexes [43], the frequency differences between the variously coordinated species are much smaller (vide infra).

NBO analysis results concerning the charge transfer (CT) interactions in the ground-state Ln(CO)_8_ species are compiled in Table 2. The Ln natural charges are all negative, indicating a predominant (CO)_8_→Ln donation in terms of transferred electrons. The number of donated and back-donated electrons was estimated by the populations of lone pair and—in the “naked” atom—empty valence orbitals of Ln. According to them, the (CO)_8_→Ln donation amounts to ca. 2 e, whereas the Ln→(CO)_8_ back-donation is less by ca. 0.5 e. The significantly smaller CT in the high-spin complexes of Sm and Eu is noteworthy. This is in agreement with the efforts in these lanthanides to approach the stable 4f^7^ configuration, thus leaving less electrons on the 5d orbitals for donor–acceptor interactions.

The energetic relation of the (CO)_8_→Ln donation and Ln→(CO)_8_ back-donation was assessed by ETS-NOCV analyses of the La, Eu, and Yb complexes. Table 3 presents the energies and characters of the most important ETS-NOCV pairs. The data demonstrate that the orbitals corresponding to Ln→(CO)_8_ back-donations have energies by one order of magnitude larger than the (CO)_8_→Ln donations. Accordingly, the energetic driving force for the formation of these complexes is the Ln→(CO)_8_ back-donation, which is similar to the anionic Ln(CO)_8_^−^ (Ln = Tm, Yb, Lu) [38] and various ionic and neutral TM–carbonyl complexes [12] reported recently. Therefore, the energetics of CT interactions show an opposite trend for the (CO)_8_→Ln vs. Ln→(CO)_8_ interactions compared to the transferred electrons (vide supra in Table 2). Another noteworthy feature in Table 3 is the smaller CT and lacking β-spin Ln→(CO)_8_ contribution in the Eu complex, in agreement with its high-spin character.

**Table 3:** Energies of the most important ETS-NOCV pairs ^1^ in the La(CO)_8_, Sm(CO)_8_, and Yb(CO)_8_ complexes from B3LYP/VTZP calculations.

Character	La		Eu		Yb
	α	β	α	β	
Ln→(CO)_8_	−922.2	−883.7	−818.4	-	−2022.9
	−343.3		−303.0		
(CO)_8_→Ln	−88.6	−88.3	−86.2	−76.5	−130.4
	−29.2	−22.1			

^1^ In kJ/mol. For La and Eu, the contributions of α and β spins are distinguished. Selected ETS-NOVC pair orbitals of La(CO)_8_ are depicted in Figure 3.

### 2.3. Identification of La(CO)_8_ in the MI-IR Spectra Assisted by DFT Calculations

As suggested by literature on related complexes, e.g., high coordination numbers up to 12 in crystals of Ln-containing compounds [44], LaO(CO)_9_^+^ [45] and ionic octa-carbonyl rare earths (Sc, Y, La, Ce, Tm, Yb, Lu) in the gaseous state detected by mass spectrometry [22,36,37,38], and the high computed stability of the latter ionic Ln(CO)_8_ species [22,36,37,38], the thermally evaporated Ln atoms likely form octa-carbonyl Ln(CO)_8_ complexes in our MI experiments in contrast to the earlier accepted hexa-carbonyl Ln(CO)_6_ [13,14,15,17,27].

Support for the above hypothesis was provided by our DFT calculations. On the basis of the recently reported good performance of the BP86 [46,47] exchange–correlation function for vibrational frequencies of Ln(CO)_8_^−^ [22,37,38] and Ln(N_2_)_x_ complexes [43], we carried out a few comparative calculations at the BP86/VTZP level. Unfortunately, the deficient SCF convergence properties of this level for several open-shell Ln(CO)_x_ systems hindered its overall application in the present study. Selected computed vibrational data are compiled in Table 4.

For validation of the BP86/VTZP level we can use the experimental information on the gaseous La(CO)_8_^−^ anion detected by mass spectrometry, and the IR active CO vibrational frequency measured by mass-selected infrared photodissociation spectroscopy [37]. Our BP86/VTZP calculations reproduced the reported O_h_ symmetry, whereas the calculated triply degenerate CO stretching mode (1907 cm^−1^) closely approaches the experimental value of 1914 cm^−1^ [37].

A similar excellent agreement was obtained for the highest-wavenumber IR bands of matrix-isolated neutral La(CO)_x_ species, albeit here the experimental frequencies have not yet been assigned to the octa-coordinated species: these are 1985 cm^−1^ [28], 1983.3 cm^−1^ [21], and the present 1992 cm^−1^ in cryogenic Ar/CO, Ne/CO, and neat CO matrices, respectively.

However, before any conclusion on the specific La(CO)_x_ species can be taken, one should have some idea about the matrix shifts. For this purpose we performed BP86/VTZP calculations on the isolated La(CO)_8_ molecule and on the La(CO)_8_Ar_8_ and La(CO)_8_(CO)_8_ model structures. In the latter molecules, the second-sphere ligands (Ar and CO, respectively) were attached to the first-sphere CO ligands along the La-C-O lines. This arrangement kept the D_4h_ symmetry of the structures (we should note that these two structures do not exactly correspond to structures in the matrix, but just serve to assess the CO^…^Ar and CO^…^CO interactions in properly small and symmetric models). Interestingly, the computed IR-active CO stretching frequencies of the latter two models differed negligibly from that of the isolated Ln(CO)_8_ (cf. Table 4), implying only a marginal influence of the cryogenic matrices on this spectral parameter. Accordingly, the excellent agreement of the BP86/VTZP frequencies with the experimental ones suggest that the La(CO)_8_ complex was formed in all three matrices.

**Figure 4:**
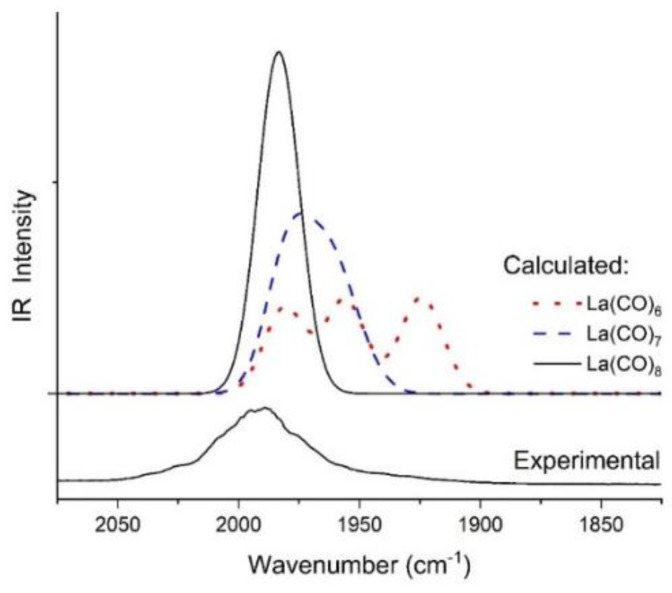
Absorption bands of ^2^La(CO)_8_, ^2^La(CO)_7_, and ^2^La(CO)_6_ from BP86/VTZP calculations (HW = 20 cm^−1^) and the experimental spectrum.

Further support for the formed La(CO)_8_ complex was provided by comparison of the computed BP86/VTZP IR spectra of octa-, hepta-, and hexa-coordinated La(CO)_x_ in the 2075–1825 cm^−1^ region in Figure 4.

At the applied model half-width (mimicking the broad bands in the matrix–isolation spectra), the highly symmetric ^2^La(CO)_8_ complex gives rise to a single intense band. The DFT calculations predicted low (C_1_) symmetry of the ^2^La(CO)_7_ complex, resulting in a multiplet of six bands lying within 34 cm^−1^ (Table S3). At the applied half-width they add up to one broad band that is slightly red-shifted from (and overlapping with) the band of ^2^La(CO)_8_. Consequently, because of this small difference in the positions of the calculated bands of ^2^La(CO)_8_ and ^2^La(CO)_7_, these two coordination forms cannot unambiguously be distinguished on the basis of the IR spectra. Nevertheless, a strong hint for Ln(CO)_8_ was provided by the relatively narrow tops of the main bands in the IR spectra of several Ln (cf. Figure 1), and was not applicable for the asymmetric Ln(CO)_7_.

The hexa-coordination case is more clear, where ^2^La(CO)_6_ produces three well-separated intense bands that differ up to 60 cm^−1^ because of the low (C_2_) symmetry. The lowest-frequency one is considerably red-shifted from ^2^La(CO)_8_ and the large separation within the triplet would easily be recognized in the experimental spectrum. In our MI-IR spectrum it is not there.

We also considered the quartet ^4^La(CO)_6_ form. This has high symmetry (O_h_) and thus would give a single intense band in this region of the IR spectrum. Its computed frequency is lower by ca. 25 cm^−1^ than that of ^2^La(CO)_8_. However, our test computations at various levels of theory consistently resulted in less stability of quartet ^4^La(CO)_6_ with respect to the doublet form (12–35 kJ/mol). Therefore, an assignment of the main band in our IR spectra to La(CO)_6_ is less likely.

BP86/VTZP calculations have been performed successfully for Eu(CO)_8_, Gd(CO)_8_, and Yb(CO)_8_, where the good agreement of the calculated frequencies with the experimental ones further supports the reliability of this theoretical level. However, due to SCF issues, the octa-coordinated complexes of the whole Ln row (except for Pm) have been calculated at the technically more stable B3LYP/VTZP level. The computations resulted generally in highly symmetric structures for Ln(CO)_8_, in agreement with the single (though broad) absorption bands in the IR spectra of most Ln. The calculated IR active CO stretching modes are listed in Table 4, where we can observe a consistent deviation from the experimental wavenumbers. Therefore, in the comparison with experimental data, uniformly scaled B3LYP frequencies were used by applying a scale factor of 0.96. These scaled B3LYP frequencies excellently reproduced the experimental ones with an average deviation of 3.7 cm^−1^ (altogether 24 bands, cf. Table 4).

The general decreasing trend in the position of the main experimental band along the Ln row (cf. Figure 1) is well-reflected in the scaled B3LYP frequencies of the IR active CO stretching vibrations of Ln(CO)_8_ species (Table 4). The decreasing trend is broken at Eu and Yb, and is well-reproduced by the DFT calculations.

**Figure 5:**
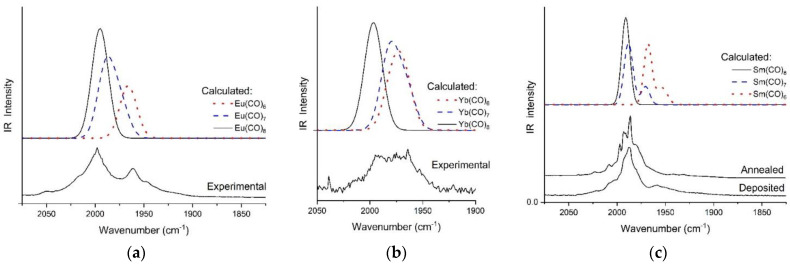
Comparison of experimental and scaled B3LYP (HW = 20 or 10 cm^−1^) IR spectra of (**a**) Eu(CO)_x_; (**b**) Yb(CO)_x_ and (**c**) Sm(CO)_x_ (calculated x = 6–8) in the CO stretching region.

Exceptionally, the main feature in the experimental spectra of Eu and Yb consisted of two intense broad bands separated by ca. 35 cm^−1^ (cf. Figure 5). The separation of these bands is in the magnitude of the calculated frequency difference between Ln(CO)_8_ and Ln(CO)_6_. In addition, in the FT-IR spectrum of Sm, a small but significant band can be recognized at 1959 cm^−1^, separated from the main band by ca. 30 cm^−1^.

Comparison of the experimental and calculated spectra of Sm, Eu, and Yb complexes in Figure 5 support the significant co-formation of octa- and hexa-coordinated species during the deposition of these Ln atoms.

The FT-IR spectrum of Sm atoms deposited with neat CO was further studied by annealing of the matrix (cf. Figure 5c): the overlapping features of the broad central band resolved partially and the growth/loss of features on the low- and higher-wavenumber side of the main band became distinguishable. On the basis of the computed spectra the resolved largest narrow peak at 1986 cm^−1^ could be assigned to Sm(CO)_7_, contrasting with the intense split band at 1992 cm^−1^ to Sm(CO)_8_. As the intensity relations of the composite main band did not change significantly upon annealing it seems that no serious conversion of Sm(CO)_7_ to Sm(CO)_8_ occurred. In contrast, the weak band at 1959 cm^−1^ assigned to Sm(CO)_6_ vanished upon annealing due to probable conversion to higher-coordinated species.

Additionally, we note the broad shoulders in most experimental IR spectra in Figure 1 that were recognized at both the low- and high-wavenumber sides of the main bands. On the basis of the calculations (Table S3 in the Supplementary Material), the features at the low-wavenumber side of the main band can likely be attributed to minor species that are lower than octa-coordination. The shoulders at the high-wavenumber side may indicate the possible formation of higher (>8) coordinated species. This hypothesis is supported by the detection of Tm(CO)_9_^−^ in the mass spectrum of Tm carbonyl anion complexes [38]. In addition, in the case of heavier Ln, the broad high-wavenumber shoulders may also include contributions from Ln(CO)_7_. In the computations on Tm(CO)_7_ and Lu(CO)_7_, a high-frequency normal mode with small but significant IR intensity was predicted (cf. Table 4 and Table S3).

## 3. Materials and Methods

### 3.1. Matrix–Isolation Spectroscopy

Details of the experimental conditions have recently been extensively reported [43]. A Displex closed-cycle helium refrigerator was used to cool a NaCl optical window mounted in a copper holder, with CaF_2_ outer windows of the vacuum shroud. For each experiment survey, IR and UV spectra were taken at 10 K to check for impurities.

Monoatomic metal vapors were generated by direct resistive heating of a metal filament or from metal powder carried in a tungsten or molybdenum Knudsen cell mounted between water-cooled electrodes in a water-cooled oven. The metals were obtained from Goodfellow as foil (0.25 mm, 99%) or powder (500 microns, 99%); all high-purity gases were obtained from Messer-Griesheim (Ar 5.7, CO 4.7). The rate of metal atom deposition was continuously monitored and controlled using an integrated quartz-crystal microbalance, with the deposition rate set such that the probability of a metal atom having another metal atom as their nearest neighbor in the matrix was of the order of 1:1000. Matrix–gas flows were mostly maintained at roughly 2 mmol/h. The matrix–isolation experiments covered the whole lanthanide row, except for the radioactive Pm.

Infrared spectra were recorded on Perkin-Elmer PE580, PE1720, or PE Paragon FT-IR spectrometers (all equipped with data-collection computers). UV-visible spectra were recorded on computer-controlled double-beam Perkin-Elmer Hitachi 320 or 330 spectrometers. IR and UV-visible spectra were taken on the same sample within a few minutes. For photochemical experiments, the light of a 1000 W Xenon (or Hg/Xe) lamp was filtered by a Schoeffel GM250 monochromator system (10 nm resolution).

### 3.2. Computational Details

The DFT computations were performed with the Gaussian09 [48] code, applying the hybrid B3LYP [49,50] exchange–correlation (XC) functional in conjunction with the quasi-relativistic small-core 4f-in-valence pseudopotentials of Ln (ECP28MWB) [51] with (14s13p10d8f6g)/(10s8p5d4f3g) contracted valence basis sets [52]. The selection of the 4f-in-valence pseudopotentials for Ln (instead of the 4f-in-core ones) was reasoned by the importance of the spin multiplicity property for the structure (and consequently for the vibrational spectra) of Ln(CO)_x_ molecules. The correlation consistent cc-pVTZ basis sets for C and O [53] extend the valence bases of the applied pseudopotentials, providing a balanced basis set for the title molecular systems. This theoretical level is denoted in the paper as B3LYP/VTZP.

In test calculations on La, Eu, Gd, and Yb complexes, the BP86 [46,47] exchange–correlation functional with the same basis sets was also applied, taking advantage of the good reproduction of the experimental vibrational frequencies by this functional [22,37,38,43].

In general, we probed two (La, Nd, Sm, Yb, Lu) or three (Ce, Pr, Eu, Gd, Tb, Dy, Ho, Er, Tm) reasonably low-energy spin states that were selected on the basis of literature data on the neutral Ln atoms [33,34,40], as compiled in Table S1 of the Supplementary Material. The spin contamination was checked carefully in the spin unrestricted calculations, where the values arisen in the SCF circles are automatically improved by the annihilation method incorporated in Gaussian09. In most cases, the obtained total spin values were in excellent agreement with the theoretical ones. However, high spin contamination was obtained for the lowest spin multiplicities of Ce, Pr, Eu, Gd, Tb, Dy, Ho, Er, and Tm (cf. Table S2), meaning that the computed data of these states (having mostly 4f^n^6s^2^ configuration) were unreliable and were therefore omitted from our discussion.

Dispersion interaction was accounted for by the D3 version of Grimme’s dispersion correction using the original D3 damping function [54]. The atomic charges and Ln orbital populations were evaluated on the basis of the Natural Bond Orbital (NBO) model [55] using the NBO 6.0 code [56,57]. Due to the deficiency of NBO 6.0 for g functions, the g polarization functions were omitted from the metal basis sets for this analysis. The ETS-NOCV (extended transition state-natural orbitals for chemical valence) analyses [58] were performed with Multiwfn 3.8 [59] on the basis of Gaussian09 checkpoint files. For visualization purposes, the GaussView 5 software [60] was applied.

## 4. Conclusions

In the present study, lanthanide carbonyl complexes, synthesized in neat CO cryogenic matrices, were investigated by infrared and UV-visible spectroscopy. In parallel, DFT computations were performed to characterize all the high-coordinated Ln(CO)_8_ and selected Ln(CO)_7_ and Ln(CO)_6_ species.

The B3LYP and (for selected Ln) BP86 computations supported the preferred formation of octa-coordinated Ln(CO)_8_ complexes in neat CO matrices. The geometry optimizations converged mostly to highly symmetric Ln(CO)_8_ structures, correlating well with the single main features in most FT-IR spectra. The scaled B3LYP vibrational frequencies reproduced the experimental wavenumbers with an average deviation of 3.7 cm^−1^.

In the cases of Sm, Eu, Tm, Yb, and Lu, contributions of hepta- and/or hexa-coordinated complexes could also be identified in the spectra. The computed structures of these complexes were less symmetric than those of Ln(CO)_8_, resulting in more than one predicted intense IR absorption. Due to the close vibrational frequencies, however, the bands partly overlap with those of Ln(CO)_8_.

The B3LYP computations extended with NBO and ETS-NOCV analyses provided important information on the bonding in the Ln carbonyl complexes. They clarified that the out-of-trend properties around the middle of the Ln row are due to the preference of these lanthanides for the 4f^7^ configuration. The complexes are based on extensive donor–acceptor interactions, from which the larger CO→Ln donation results in the Ln atoms getting negatively charged. However, from an energetic point of view, the Ln→CO back-donations are much more efficient, as observed recently for anionic Ln(CO)_8_^−^ (Ln = Tm, Yb, Lu) [38] and various ionic and neutral TM–carbonyl complexes [12].

## Figures and Tables

**Figure 3:**
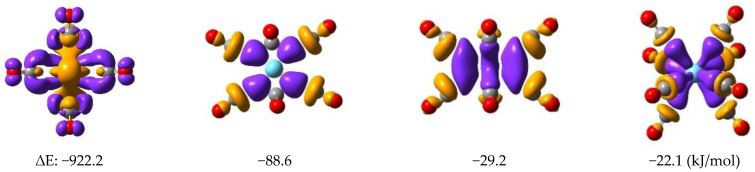
Plots of the density isosurfaces of characteristic NOCV pairs of La(CO)_8_. The colors yellow and violet mean charge depletion and accumulation, respectively. The atomic colors are as follows: cyan, La; gray, C; red, O.

**Table 1:** Selected properties ^1^ of Ln(CO)_8_ complexes from B3LYP/VTZP computations.

Ln	m		Pop_Ln_		Sym	Ln-C	C≡O
		6s	4f	5d			
La	2	0.38	0.09	2.98	D_4h_	2.703	1.136
Ce	3	0.40	1.08	3.07	D_4h_	2.651	1.136
Pr	4	0.40	2.08	3.10	D_4h_	2.627	1.136
Nd	5	0.41	3.06	3.10	D_4h_	2.606	1.136
Sm	9	0.39	5.83	1.99	O_h_	2.692	1.135
Eu	10	0.39	6.86	1.95	O_h_	2.704	1.135
Gd	9	0.72	7.00	2.75	D_4h_	2.607	1.136
Tb	8	1.00	7.42	3.16	D_2d_	2.543	1.137
Dy	7	0.93	8.49	3.16	C_s_	2.511, 2.515	1.137
Ho	6	0.44	10.10	3.18	D_4h_	2.494	1.137
Er	5	0.44	11.01	3.15	D_4h_	2.479	1.138
Tm	4	0.44	12.02	3.14	D_4h_	2.464	1.137
Yb	1	0.43	13.77	2.37	D_4d_	2.528	1.136
Lu	2	0.44	14.00	2.95	D_2h_	2.447	1.138

^1^ The properties include spin multiplicity (m); natural populations of the Ln valence orbitals from NBO analysis (Pop_Ln_, e); symmetry of the structure; Ln-C and C≡O bond distances (Å).

**Table 2:** NBO analysis results on the charge transfer interactions ^1^ of Ln(CO)_8_ complexes from B3LYP/VTZP calculations.

Ln	q_Ln_	CT	
		(CO)_8_→Ln	Ln→(CO)_8_
La	−0.46	1.99	1.53
Ce	−0.54	2.12	1.58
Pr	−0.58	2.14	1.56
Nd	−0.57	2.15	1.58
Sm	−0.21	1.63	1.42
Eu	−0.20	1.58	1.38
Gd	−0.47	1.97	1.50
Tb	−0.59	1.99	1.61
Dy	−0.59	1.99	1.62
Ho	−0.63	2.26	1.63
Er	−0.61	2.26	1.66
Tm	−0.60	2.28	1.68
Yb	−0.57	2.00	1.43
Lu	−0.39	2.12	1.72

^1^ Natural charge of Ln (q, e); charge transfer between Ln and the (CO)_8_ fragments (CT, e) derived from the populations of lone pair (LP) and—in the “naked” atom—empty valence (LV) orbitals of Ln.

**Table 4:** Characteristic vibrational data computed at the BP86/VTZP and B3LYP/VTZP levels and the positions of the main bands in the IR spectra ^1^.

Ln(CO)x	BP86	B3LYP	B3LYP Scaled ^2^	Experimental ^3^
La(CO)_8_	2 × 1984 (3025), 1982 (2605)	2 × 2075 (3542), 2072 (3236)	1991	1992; 1985 [28]; 1983.3 [21]
La(CO)_8_^−^	3 × 1907 (3483)			1914 [37]
La(CO)_8_Ar_8_	2 × 1984 (3144), 1982 (2654)			
La(CO)_8_(CO)_8_	2 × 1985 (3156), 1984 (2618)			
Ce(CO)_8_		2077 (3352), 2 × 2070 (3221)	1990	1992; 1985.1 [20]
Pr(CO)_8_		2 × 2075 (3212), 2068 (3158)	1989	1988; 1989 [15]
Nd(CO)_8_		2074 (3322), 2 × 2071 (3132)	1989	1987; 1990 [14]
Sm(CO)_8_		3 × 2079 (3425)	1996	1992 ^4^
Sm(CO)_7_		2 × 2072 (2972), 2068 (1914)	1989, 1985	1986, 1981
Sm(CO)_6_		2 × 2050 (3593)	1967	1959
Eu(CO)_8_	3 × 1993 (2589)	3 × 2078 (3562)	1995	1999; 2000 [15]
Eu(CO)_6_	2 × 1970 (2717), 1957 (1289)	2 × 2049 (4400), 2043 (1771)	1967	1960
Gd(CO)_8_	2 × 1987 (2385), 1979 (2930)	2 × 2076 (2957), 2066 (3493)	1993, 1983	1989, 1985; 1986 [15]
Tb(CO)_8_		2 × 2071 (3105), 2069 (2965)	1987	1985
Dy(CO)_8_		2 × 2070 (2642), 2068 (2912)	1986	1983
Ho(CO)_8_		2067 (3170), 2 × 2065 (2982)	1983	1980 ^5^; 1982 [15]
Er(CO)_8_		2066 (3167), 2 × 2064 (2986)	1982	1980
Tm(CO)_8_		3 × 2063 (3100)	1980	1977
Tm(CO)_7_		2070 (1505), 2059 (1461) 2054 (3196), 2048 (2595)	1987, 1969	1991, 1971
Yb(CO)_8_	3 × 1992 (2310)	2081 (3105), 2 × 2079 (3025)	1997	1990; 1995 and 2008 [14]
Yb(CO)_6_	1975 (2941), 2 × 1964 (1675)	2061 (3841), 2 × 2050 (2199)	1973	1967
Lu(CO)_8_		2063 (3104), 2 × 2062 (2960)	1980	1975
Lu(CO)_7_		2074 (1290), 2059 (1391), 2054 (3216), 2047 (1459), 2047 (1182)	1991, 1972	1990, 1965

^1^ Vibrational frequencies in cm^−1^, computed intensities in parenthesis in km/mol. ^2^ Using scale factor 0.96. For the close lying computed frequencies, the average wavenumber value was considered. ^3^ Present MI-IR measurements in CO matrix and literature data including the references. ^4^ Reported preliminarily as 1987 cm^−1^ in Ref. [17]. ^5^ Reported preliminary as 1981 cm^−1^ in Ref. [27].

## Data Availability

All data analyzed in the study are included in this article. If more information is needed, it can be available on request from the corresponding author.

## Supplementary Materials

The following supporting information can be downloaded at: https://www.mdpi.com/article/10.3390/molecules28135043/s1.

## Sample Availability

Not available.
