# Supplementary material for: A DFT and Matrix–Isolation IR/UV-Visible Study of High-Coordinated Lanthanide-CO Complexes
Source: Molecules. 2023 Jun 28;28(13):5043. doi: 10.3390/molecules28135043 (PMC10343325; doi:10.3390/molecules28135043)
Supplement: Supplementary file 1 [file molecules-28-05043-s001.zip › molecules-2441719-supplementary.pdf]

Supplementary Material

A DFT and matrix-isolation IR/UV-visible study of high-coordinated lanthanide-CO complexes

Attila Kovács,[a] Werner Klotzbücher[b]

[a] *European Commission, Joint Research Centre (JRC), Karlsruhe, Germany*

Mail: P.O. Box 2340, 76125 Karlsruhe, Germany; e-mail: attila.kovacs@ec.europa.eu

[b] *Former Max Planck Institute for Radiation Chemistry, 45470 Mülheim a.d. Ruhr,*

Stiftstrasse 34-36, Germany; e-mail: werner.klotzbuecher@cec.mpg.de

Content:

Table S1. Relative stability of the significant high-spin and low-spin states of neutral Ln atoms.
Table S2. Selected computed properties of $Ln(CO)_8$ complexes.
Table S3. Main computed properties[a] of selected $Ln(CO)_8$, $Ln(CO)_7$ and $Ln(CO)_6$ complexes.
Table S4. Absorption bands (nm) of $Ln(CO)_x$ species isolated in pure CO or CO/Ar (noted) matrix observed in the UV-visible spectra.
Characteristic UV-visible and FT-IR spectra of all the deposited Ln atoms.
Cartesian coordinates of the optimized structures.

Table S1. Relative stability of the significant high-spin and low-spin states of neutral Ln atoms.

Ln	m	Term	Config	ΔE(cm^{-1})	ΔE(kJ/mol)
La	2	$^{2}D_{3/2}$	$6s^{2}5d^{1}$	0.0	0.0
	4	$^{4}F_{3/2}$	$6s^{1}5d^{2}$	2668.188	31.9
Ce	1	$^{1}G_{4}$	$6s^{2}5d^{1}4f^{1}$	0.0	0.0
	3	$^{3}F_{2}$	$6s^{2}5d^{1}4f^{1}$	228.849	2.7
	5	$^{5}H_{3}$	$6s^{1}5d^{2}4f^{1}$	2369.068	28.3
Pr	2	$^{2}H_{9/2}$	$6s^{2}5d^{1}4f^{2}$	5822.87	69.7
	4	$^{4}I_{9/2}$	$6s^{2}4f^{3}$	0.0	0.0
	6	$^{6}L_{11/2}$	$6s^{1}5d^{2}4f^{2}$	6714.22	80.3
Nd	5	$^{5}I_{4}$	$6s^{2}4f^{4}$	0.0	0.0
	7	$^{7}L_{5}$	$6s^{1}5d^{1}4f^{4}$	8475.355	101.4
Sm	7	$^{7}F_{0}$	$6s^{2}4f^{6}$	0.0	0.0
	9	$^{9}H_{1}$	$6s^{1}5d^{1}4f^{6}$	10801.10	129.2
Eu	6	$^{6}D_{9/2}$	$6s^{1}5d^{1}4f^{7}$	19273.24	230.6
	8	$^{8}S_{7/2}$	$6s^{2}4f^{7}$	0.0	0.0
	10	$^{10}D_{5/2}$	$6s^{1}5d^{1}4f^{7}$	12923.72	154.6
Gd	7	$^{7}D_{5}$	$6s^{2}5d^{1}4f^{7}$	6976.508	83.5
	9	$^{9}D_{2}$	$6s^{2}5d^{1}4f^{7}$	0.0	0.0
	11	$^{11}F_{2}$	$6s^{1}5d^{2}4f^{7}$	6378.146	76.3
Tb	6	$^{6}H_{15/2}$	$6s^{2}4f^{9}$	0.0	0.0
	8	$^{8}G_{13/2}$	$6s^{2}5d^{1}4f^{8}$	285.500	3.4
	10	$^{10}G_{15/2}$	$6s^{1}5d^{2}4f^{8}$	8190.465	98.0
Dy	5	$^{5}I_{8}$	$6s^{2}4f^{10}$	0.0	0.0
	7	$^{7}H_{8}$	$6s^{2}5d^{1}4f^{9}$	7565.60	90.5
	9	[b]			
Ho	4	$^{4}I_{15/2}$	$6s^{2}4f^{11}$		
	6	[b]			
	8	[b]			
Er	3	$^{3}H_{6}$	$6s^{2}4f^{12}$		
	5	[b]			
	7	[b]			
Tm	2	$^{2}F_{7/2}$	$6s^{2}4f^{13}$		
	4	[b]			
	6	[b]			
Yb	1	$^{1}S_{0}$	$6s^{2}4f^{14}$	0.0	0.0
	3	$^{3}D_{1}$	$6s^{1}5d^{1}4f^{14}$	24489.102	193.0
Lu	2	$^{2}D_{3/2}$	$6s^{2}5d^{1}4f^{14}$	0.0	0.0
	4	$^{4}F_{3/2}$	$6s^{1}5d^{2}4f^{14}$	18851.31	225.5

[a]From Martin, W.C.; Zalubas, R.; Hagan, L. *Atomic energy levels— The rare-earth elements. The spectra of Lanthanum, Cerium, Praseodymium, Neodymium, Promethium, Samarium, Europium, Gadolinium, Terbium, Dysprosium, Holmium, Erbium, Thulium, Ytterbium, and Lutetium*; National Bureau of Standards, U.S. Department of Commerce: Washington, US, 1978.
[b]The character of these (lowest-energy) high-spin states of Dy, Ho, Er and Tm is not available because in their atomic spectra numerous low-energy lines are left unassigned.

Table S2. Selected computed properties[a] of $Ln(CO)_8$ complexes.

Ln	m	$\langle S^2 \rangle$	Sym	ΔE	Ln-C	C≡O	$\langle S^2 \rangle_{theor}$
La	2	0.75	D_{4h}	0.0	2.703	1.136	0.75
	4	3.75	D_{4d}	82.8	2.719	1.138	3.75
Ce	1	1.00	D_{4h}	5.7	2.654	1.136	0.00
	3	2.00	D_{4h}	0.0	2.651	1.136	2.00
	5	6.00	C_2	78.6	2.672-2.679	1.137-1.138	6.00
Pr	2	1.75	D_{4h}	10.2	2.628	1.136	0.75
	4	3.75	D_{4h}	0.0	2.627	1.136	3.75
	6	8.75	O_h	49.1	2.683	1.136	8.75
Nd	5	6.00	D_{4h}	0.0	2.606	1.136	6.00
	7	12.00	C_{2h}	43.2	2.667, 2.726	1.137, 1.132	12.00
Sm	7	12.39	C_1	18.5	2.608-2.647	1.135-1.137	12.00
	9	20.00	O_h	0.0	2.692	1.135	20.00
Eu	6	10.76	O_h	28.7	2.721	1.135	8.75
	8	16.76	D_{4d}	15.3	2.673	1.135	15.75
	10	24.76	O_h	0.0	2.704	1.135	24.75
Gd	7	13.07	C_2	14.0	2.608-2.609	1.136	12.00
	9	20.32	D_{4h}	0.0	2.607	1.136	20.00
	11	30.21	C_1	179.0	2.445-2.749	1.132-1.146	30.00
Tb	6	10.37	C_1	SCF	-	-	8.75
	8	16.37	D_{2d}	0.0	2.543	1.137	15.75
	10	24.75	D_{2d}	125.7	2.518	1.137	24.75
Dy	5	7.55	C_2	10.1	2.514-2.520	1.137	6.00
	7	12.55	C_s	0.0	2.511, 2.515	1.137	12.00
	9	20.54	C_1	105.3	2.510-2.575	1.138-1.140	20.00
Ho	4	4.76	C_{4h}	0.4	2.494	1.138	3.75
	6	8.76	D_{4h}	0.0	2.494	1.137	8.75
	8	15.76	C_2	99.0	2.515-2.518	1.139	15.75
Er	3	3.01	C_1	-8.3	2.478-2.489	1.137	2.00
	5	6.01	D_{4h}	0.0	2.479	1.138	6.01
	7	12.01	C_1	89.7	2.496-2.505	1.139-1.140	12.01
Tm	2	1.75	D_{4h}	0.2	2.464	1.137	0.75
	4	3.75	D_{4h}	0.0	2.464	1.137	3.75
	6	8.75	C_1	95.4	2.480-2.486	1.139-1.140	8.75
Yb	1	-	D_{4d}	0.0	2.528	1.136	-
	3	2.01	O_h	10.0	2.584	1.136	2.00
Lu	2	0.75	D_{2h}	0.0	2.447	1.138	0.75
	4	3.75	D_{4d}	99.5	2.454	1.140	3.75

[a]The properties include: spin multiplicity (m); total spin ($\langle S^2 \rangle$) after annihilation, symmetry of the structure; relative energy of the spin states (kJ/mol); Ln-C and C≡O bond distances (Å), theoretical total spin $\langle S^2 \rangle_{theor}$.

Table S3. Main computed properties[a] of selected $Ln(CO)_8$, $Ln(CO)_7$ and $Ln(CO)_6$ complexes.

$Ln(CO)_x$	m	Sym	ΔE	Ln-C	C≡O	Unscaled frequencies (significant IR intensities)
$La(CO)_8$	4	D_{4d}	82.8	2.719	1.138	2x2038 (4485), 2091 (2373)
$La(CO)_8$	*4*	*D_{4d}*	*73.1*	*2.687*	*1.151*	*2x1962 (3193), 2001 (2089)*
$La(CO)_7$	2	C_s	0.0	2.590-2.715	1.136-1.141	2038 (1082), 2051 (1564), 2054 (1510), 2064 (1214), 2072 (2316), 2075 (1886)
	4	C_{3v}	59.3	2.639-2.844	1.133-1.142	2x1995 (1836), 2043 (2044), 2x2064 (2231), 2081 (1518)
$La(CO)_7$	*2*	*C_1*	*0.0*	*2.540-2.689*	*1.149-1.155*	*1949 (689), 1959 (1118), 1961 (1028), 1967 (1122), 1976 (2225), 1983 (1762)*
	4	*C_{3v}*	*62.3*	*2.623-2.769*	*1.148-1.155*	*2x1929 (1150), 1957 (1057), 2x1972 (1948), 1983 (2001)*
$La(CO)_6$	2	C_2	0.0	2.587-2.729	1.136-1.142	2017 (1731), 2024 (1856), 2055 (3065), 2070 (2150)
	4	O_h	12.1	2.677	1.138	3x2041 (3816)
$La(CO)_6$	*2*	*C_2*	*0.0*	*2.519-2.725*	*1.149-1.157*	*1922 (1368), 1927 (1159), 1956 (2351), 1981 (2156)*
	4	*O_h*	*27.5*	*2.652*	*1.152*	*3x1958 (3028)*
Ce	5	C_2	78.6	2.672-2.679	1.137-1.138	2006 (304), 2040 (3948), 2042 (3872), 2090 (2315)
$Pr(CO)_8$	6	O_h	49.1	2.683	1.136	3x2074 (3194)
$Pr(CO)_7$	4	C_s	0.0	2.516-2.646	1.136-1.141	2037 (1051), 2050 (1453), 2051 (1616), 2065 (1299), 2072 (1549), 2075 (2104)
	6	C_1	40.2	2.628-2.677	1.136-1.139	2x2008 (127), 2038 (371), 2x2057 (3441), 2058 (1808)
$Pr(CO)_6$	4	C_2	0.0	2.511-2.678	1.135-1.142	2014 (1598), 2020 (1871), 2053 (2891), 2074 (2089)
	6	D_{4h}	8.3	2.600-2.606	1.138	2040 (3620), 2040 (3713), 2040 (3715)
$Nd(CO)_8$	7	C_{2h}	43.2	2.667-2.726	1.132-1.137	2053 (658), 2x2065 (3345), 2102 (2490)
$Nd(CO)_7$	5	C_1	0.0	2.502-2.618	1.136-1.141	2074 (1636), 2071 (2063), 2063 (1143), 2052 (1527), 2050 (1640), 2037 (1085)
	7	C_{3v}	34.7	2.642-2.681	1.136-1.139	2x2024 (214), 2047 (513), 2x2062 (3367), 2063 (1618)
$Nd(CO)_6$	5	C_s	0.0	2.463-2.573	1.139-1.144	2053 (1941), 2049 (3535), 2017 (1372), 2011 (904)
	7	O_h	14.7	2.580	1.138	3x2040 (3704)
$Sm(CO)_8$	7	C_1	18.5	2.608-2.647	1.135-1.137	2066 (2393), 2070 (1508), 2072 (2502), 2075 (472, 2076 (1760), 2079 (1345)
$Sm(CO)_7$	9	C_{3v}	0.0	2.625-2.689	1.134-1.137	2x2072 (2972), 2068 (1914), 2054 (643), 2x2052 (777)
	7	C_1	19.2	2.614-2.655	1.136-1.137	2x2069 (2655), 2066 (2313), 2060 (316), 2x2055 (937)

$Sm(CO)_6$	9	C_{2v}	0.0	2.593-2.654	1.137-1.140	2x2050 (3593), 2036 (1641), 2030 (819)
	7	C_{2v}	12.1	2.626-2.643	1.137-1.138	2062 (4305), 2052 (2207), 2050 (2391)
$Eu(CO)_8$	8	D_{4d}	15.3	2.673	1.135	2x2084 (3205), 2087 (3566)
$Eu(CO)_8$	*8*	*D_{4d}*	*0.0*	*2.570*	*1.149*	*1996 (2778), 2x1995 (2457)*
$Eu(CO)_7$	8	C_1	18.5	2.639-2.691	1.135-1.137	2050 (1226), 2055 (812), 2057 (2612), 2059 (2207), 2067 (1945), 2072 (2479)
	10	C_{3v}	0.0	2.634-2.683	1.135-1.137	2x2073 (2589), 2068 (2321), 2057 (615), 2x2055 (1206)
$Eu(CO)_7$	*8*	*C_s*	*13.2*	*2.532-2.569*	*1.150-1.152*	*1966 (393), 1974 (1329), 1982 (2498), 1984 (28149*
	10	*C_{3v}*	*0.0*	*2.546-2.606*	*1.149-1.151*	*2x1988 (2300), 1983 (2083), 2x1972 (470)*
$Eu(CO)_6$	8	C_{2v}	10.6	2.594-2.667	1.137-1.140	2026 (952), 2029 (1864), 2049 (4580), 2054 (2505)
	10	D_{3d}	0.0	2.651	1.137	2x2049 (4400), 2043 (1771)
$Eu(CO)_6$	*8*	C_s	3.4	2.480-2.585	1.152-1.156	1964 (2060), 1962 (3252), 1946 (718), 1936 (830)
	10	C_{3v}	0.0	2.570, 2.582	1.151, 1.152	2x1970 (2717), 1957 (1289), 2x1947 (321)
$Gd(CO)_8$	11	C_1	179.0	2.445-2.749	1.132-1.146	1922 (1063), 1950 (1209), 2000 (2642), 2021 (1763), 2062 (1889), 2077 (833), 2096 (1110)
$Gd(CO)_8$	*11*	*C_1*	*209.7*	*2.463-2.640*	*1.149-1.159*	*1832 (818), 1862 (943), 1902 (1492), 1929 (1401), 1958 (1267), 1961 (680), 1985 (1139)*
$Gd(CO)_7$	9	C_s	0.0	2.527-2.611	1.134-1.139	2086 (1303), 2079 (1369), 2060 (1192, 2056 (2258), 2050 (1434), 2047 (1303)
	11	C_{3v}	140.3	2.439-2.822	1.131-1.146	2x1922 (2137), 2015 (2099), 2069 (1615), 2109 (569), 2158 (270)
$Gd(CO)_7$	*9*	*C_s*	*0.0*	*2.479-2.581*	*1.148-1.153*	*1995 (1152), 1988 (1223), 1971 (1588), 1968 (1242), 1962 (1072), 1957 (904)*
	11	*C_s*	*177.7*	*2.366-2.696*	*1.146-1.167*	*1816 (750), 1904 (2386), 1905 (2419), 1982 (1356), 1999 (820)*
$Gd(CO)_6$	9	C_s	0.0	2.490-2.604	1.136-1.142	2x2025 (1500), 2051 (3801), 2073 (1056)
	11	O_h	88.3	2.498	1.139	3x2001 (3452)
$Gd(CO)_6$	*9*	*C_s*	*0.0*	*2.442-2.574*	*1.150-1.157*	*1978 (858), 1961 (3131), 1956 (456), 1938 (654), 1934 (1142)*
	11	*O_h*	*133.0*	*2.481*	*1.154*	*3x1894 (2465)*
$Tb(CO)_8$	10	D_{2d}	125.7	2.518	1.137	2x2049 (999), 2x2065 (1967), 2077 (2834)
$Dy(CO)_8$	9	C_1	105.3	2.510-2.575	1.138-1.140	2026 (3637), 2027 (3746), 2039 (615), 2042 (667), 2089 (1804)
$Ho(CO)_8$	8	C_2	99.0	2.515-2.518	1.139	2024 (4285), 2025 (4273), 2087 (1797)
$Ho(CO)_7$	6	C_1	0.0	2.426-2.481	1.136-1.140	2074 (1435), 2061 (1382), 2057 (2944), 2049 (1424), 2048 (1154)
	8	C_3	68.6	2.433-2.622	1.134-1.145	2080 (1267), 2x2067 (1830), 2044 (1512), 2x1978 (1920)

$Ho(CO)_6$	6	C_s	0.0	2.381-2.440	1.139-1.143	2049 (2264), 2046 (3282), 2027 (1609)
	8	D_{3d}	9.7	2.448	1.139	3x2038 (3425)
$Er(CO)_8$	7	C_1	89.7	2.496-2.505	1.139-1.140	2021 (4363), 2021 (4373) 2083 (1807)
$Tm(CO)_8$	6	C_1	95.4	2.480-2.486	1.139-1.140	2022 (4272), 2022 (4268), 2084 (1784)
$Tm(CO)_7$	4	C_1	0.0	2.404-2.441	1.137-1.140	2070 (1505), 2059 (1461), 2054 (3196), 2048 (1289), 2047 (1306)
	6	C_s	74.9	2.400-2.558	1.135-1.146	1966 (1852), 1977 (1954), 2043 (1375), 2064 (1948), 2065 (1750), 2069 (1423)
$Tm(CO)_6$	4	C_s	0.0	2.361-2.409	1.140-1.143	2046 (2359), 2043 (2809), 2028 (1707), 2018 (904)
	6	C_{2h}	5.5	2.411-2.417	1.139	2x2034 (3372), 2035 (3385)
$Yb(CO)_8$	3	O_h	10.0	2.584	1.136	3x2067 (3529)
$Yb(CO)_8$	*3*	*D_4*	*29.9*	*2.478*	*1.150*	*2x1982 (2586), 1976 (2625)*
$Yb(CO)_7$	1	C_s	5.0	2.504-2.531	1.136-1.138	2049 (809), 2049 (1433), 2052 (862), 2060 (2668), 2064 (2387), 2067 (1974)
	3	C_{3v}	0.0	2.503-2.530	1.136-1.138	2x2065 (2120), 2062 (2537), 2050 (677), 2x2047 (1415)
$Yb(CO)_7$	*1*	*C_s*	*6.0*	*2.423-2.446*	*1.150-1.152*	*1983 (1531), 1982 (2217), 1979 (2166), 1966 (833)*
	3	*C_{3v}*	*0.0*	*2.423-2.444*	*1.150-1.152*	*2x1984 (1588), 1982 (2207), 2x1967 (941)*
$Yb(CO)_6$	1	D_{3h}	0.0	2.488	1.138	2061 (3841), 2x2050 (2199)
	3	D_{3d}	16.7	2.508	1.138	2035 (2238), 2x2040 (4238)
$Yb(CO)_6$	*1*	D_{3h}	0.0	2.417	1.153	1975 (2941), 2x1964 (1675)
	3	C_{3v}	16.9	2.424, 2.437	1.152, 1.154	2x1964 (2439), 1956 (1392), 2x1942 (418)
$Lu(CO)_8$	4	D_{4d}	99.5	2.454	1.140	2085 (1727), 2x2019 (4309)
$Lu(CO)_7$	2	C_{2v}	0.0	2.373-2.407	1.136-1.140	2074 (1290), 2059 (1391), 2054 (3216), 2047 (1459), 2047 (1182)
	4	C_{3v}	77.1	2.376-2.525	1.135-1.145	2x1967 (1877), 2043 (1290), 2064 (1824), 2067 (1436)
$Lu(CO)_6$	2	C_{2v}	0.0	2.334-2.376	1.140-1.143	2049 (2276), 2043 (3176), 2028 (1739), 2015 (1032)
	4	O_h	2.8	2.382	1.140	3x2034 (3329)

[a]The majority of the presented data have been computed at the B3LYP/TZP level. ***BP86/TZP results are given in italics.***

Table S4. Absorption bands (nm) of Ln(CO)$_x$ species isolated in pure CO or CO/Ar (noted) matrix observed in the UV-visible spectra.[a]

Ln	At deposition	After irradiation
La	450 s, br HW = 70 nm	-
Ce	418 s, br HW = 120 nm	375-465 vbr
	(576, 653, 719w)	
Pr	412 s, br HW = 70 nm	vanished
Pr CO/Ar	406 m, br HW = 110 nm	406 s
	(450-550 atomic features)	
Nd	415 s, br HW = 100 nm	vanished
Sm	408 s, br HW = 70 nm	408 w
	(320 – 580 atomic features)	
Eu	532 m, br HW = 180 nm	vanished
Eu CO/Ar	529 m, br; 485 m, br	
	529 remains upon tempering	
Gd	399 s, br HW = 50 nm	vanished
Tb	393 s, br HW = 50 nm	393 w
Dy	387 s, vbr HW = 100 nm	
Dy CO/Ar	387	387 m
	(240 - 475 atomic features)	
Ho	387 s, br HW = 80 nm	387 m, br
Er	388 s, br HW = 70 nm	388 m, br, 544 w
	(330 – 485 atomic features)	
Tm	382 m, br HW = 80 nm	vanished
	(330 – 550 atomic features)	
Yb	(Only strong atomic features)	468 m, br
Lu	385 s, br HW = 80 nm	vanished
	521 m, br; 582 w, br	
	(No atomic features !)	

[a]All molecular UV spectra are fairly broad. The abbreviations s, br, vbr, w and sh mean strong, broad, very broad, weak and shoulder, respectively. HW = indicative half-width

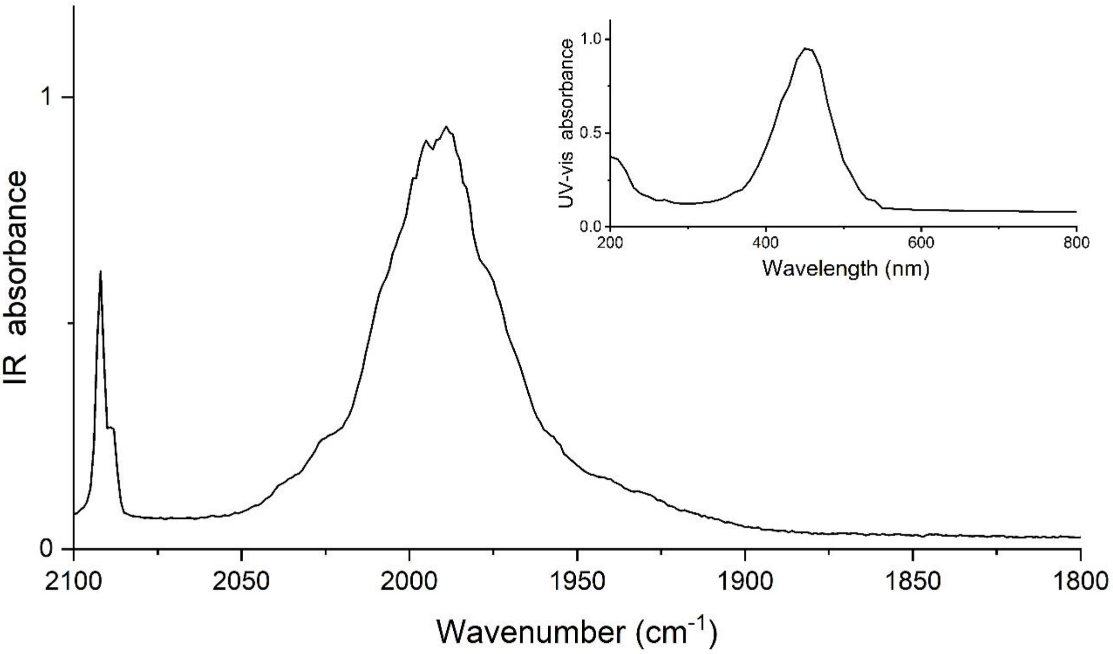

Figure S1. Characteristic UV-visible and FT-IR spectra of all the deposited Ln atoms: FT-IR spectrum of the cocondensation of lanthanum atoms with neat CO at 10 K with strong, splitted band centered at 1992 cm^{-1}. Note the band of isotopic CO at 2091/2088 cm^{-1}. Insert: UV-vis spectrum showing a strong single band centered at 450 nm with half-width of 70 nm.

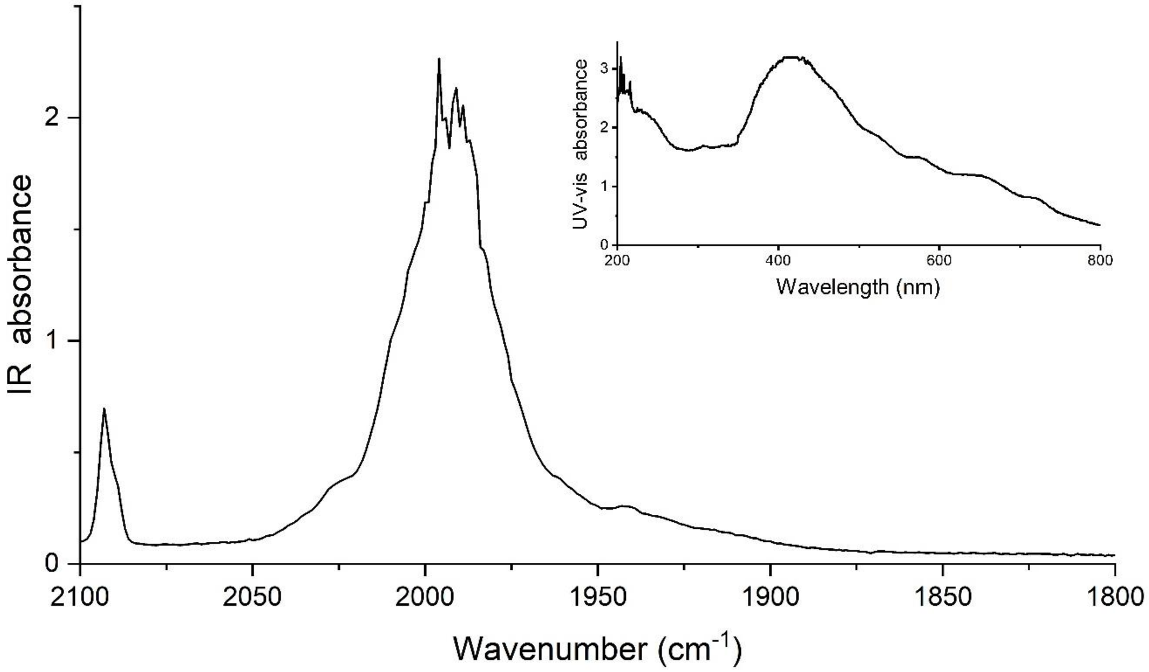

Figure S2. FT-IR spectrum of the cocondensation of cerium atoms with neat CO at 10 K with strong, splitted band centered at 1992 cm^{-1}.
Insert: UV-vis spectrum of the condensation of cerium atoms with neat CO at 10 K, with strong band centered at 418 nm.

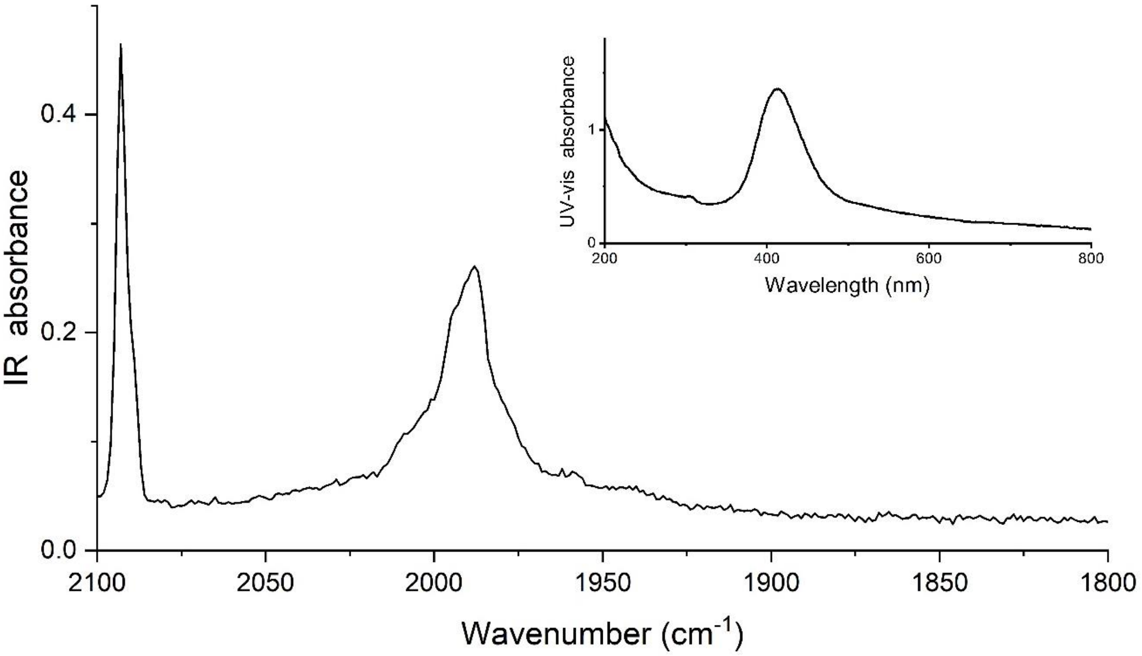

(**a**)

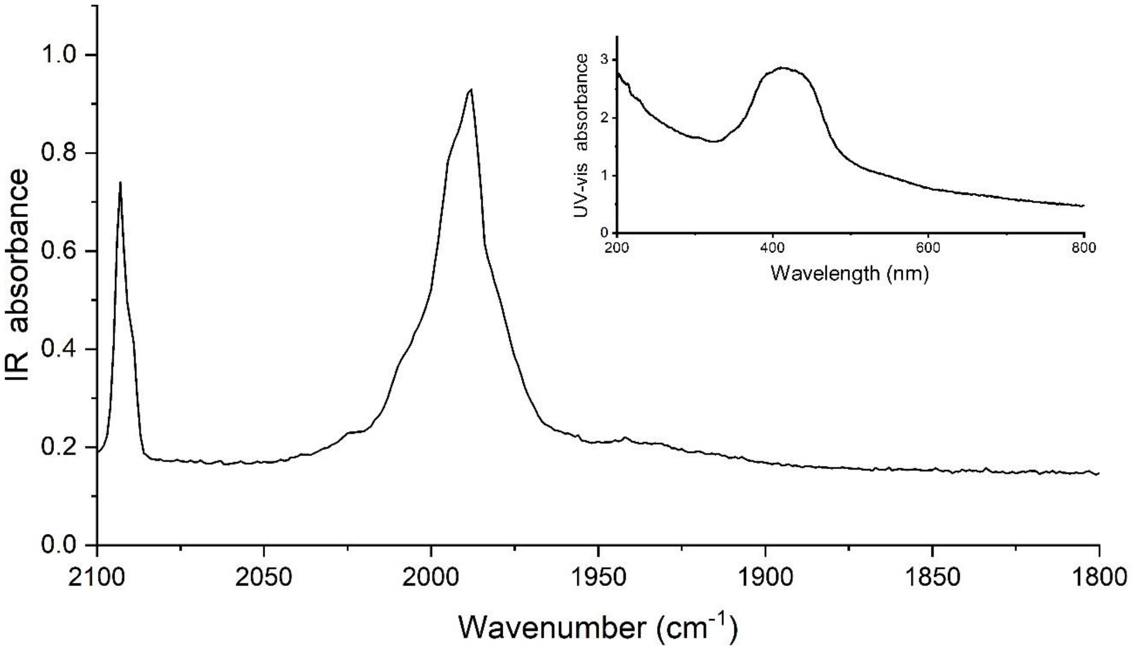

(**b**)

Figure S3. (a) FT-IR spectrum of the cocondensation of praseodymium atoms with neat CO at 10 K with medium band centered at 1988 cm^{-1}.
Insert: UV-vis spectrum of the condensation of praseodymium atoms with neat CO at 10 K, with strong band centered at 412 nm.
(**b**) FT-IR spectrum of the cocondensation of neodymium atoms with neat CO at 10 K with strong band centered at 1987 cm^{-1}.
Insert: UV-vis spectrum of the condensation of neodymium atoms with neat CO at 10 K, with strong band centered at 415 nm.

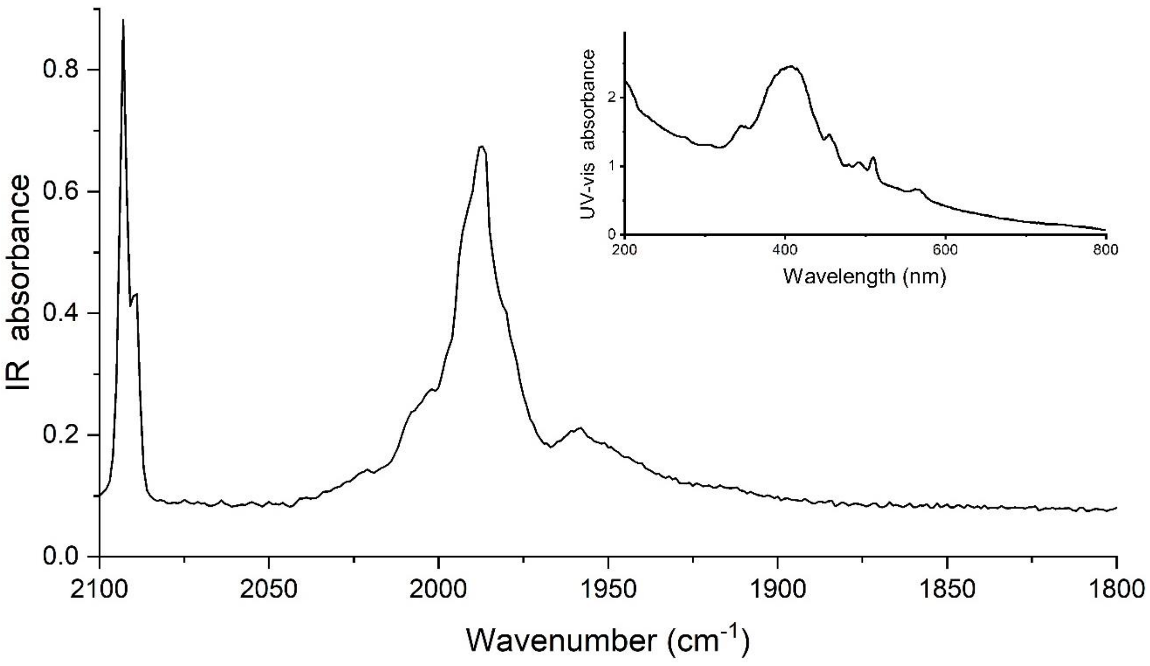

Figure S4. FT-IR spectrum of the cocondensation of samarium atoms with neat CO at 10 K with strong band centered at 1988 cm^{-1} and weak further features.
Insert: UV-vis spectrum of the condensation of samarium atoms with neat CO at 10 K, with weak atomic features and strong band centered at 408 nm.

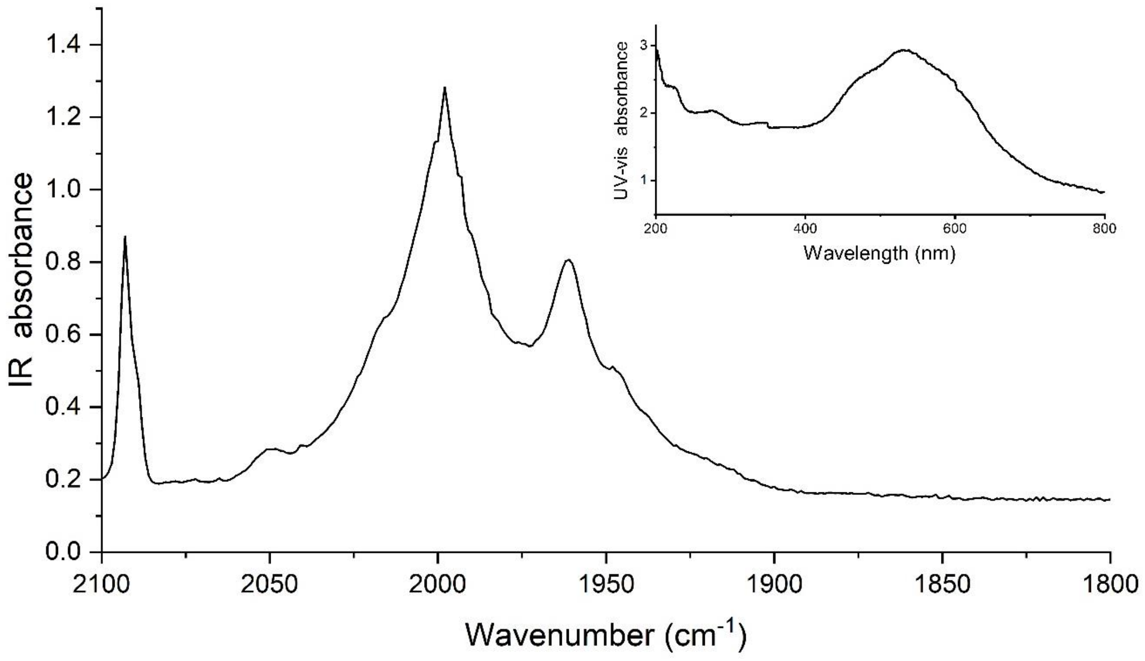

Figure S5. FT-IR spectrum of the cocondensation of europium atoms with neat CO at 10 K with two strong bands centered at 1999 and 1960 cm^{-1} as well as weaker features.
Insert: UV-vis spectrum of the condensation of europium atoms with neat CO at 10 K, with broad band centered at 532 nm.

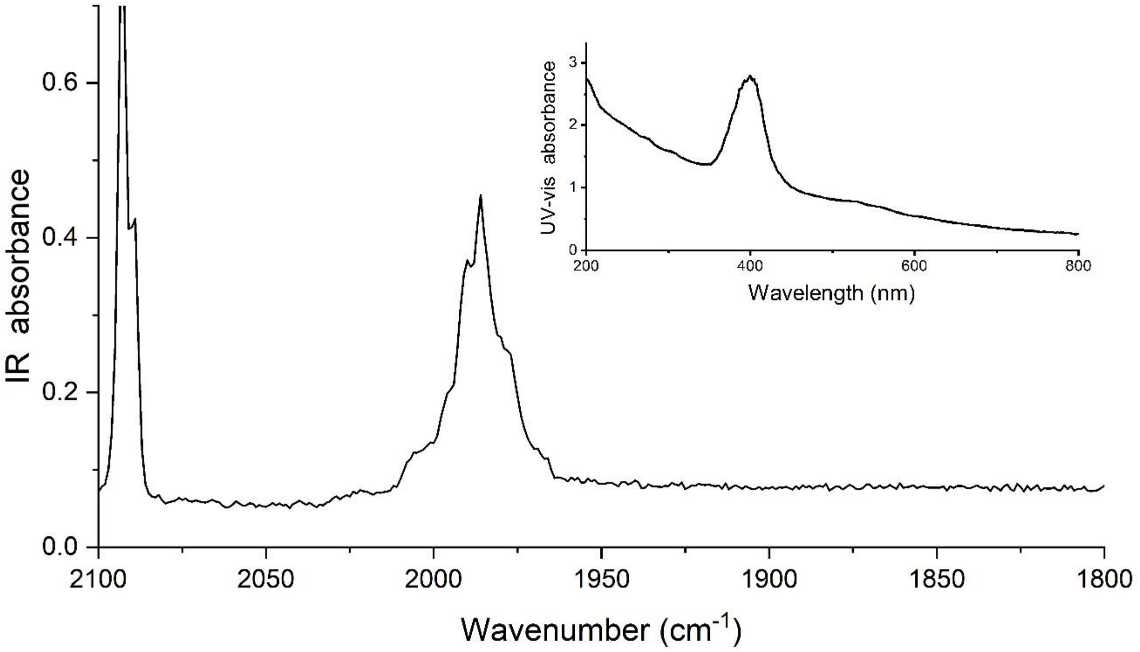

Figure S6. FT-IR spectrum of the cocondensation of gadolinium atoms with neat CO at 10 K with strong splitted band centered at 1986 cm^{-1}.
Insert: UV-vis spectrum of the condensation of gadolinium atoms with neat CO at 10 K, with broad band centered at 399 nm.

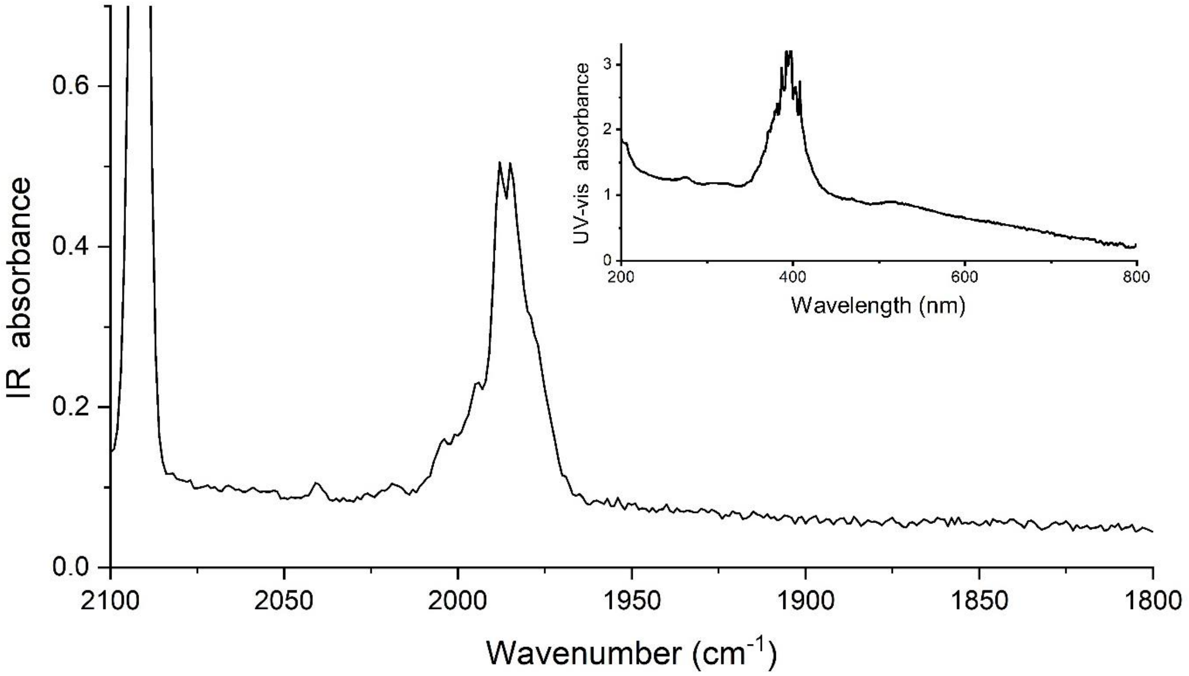

Figure S7. FT-IR spectrum of the cocondensation of terbium atoms with neat CO at 10 K with strong splitted band centered at 1985 cm^{-1}.
Insert: UV-vis spectrum of the condensation of terbium atoms with neat CO at 10 K, with broad band centered at 393 nm overlapping with spectral features of unreacted metal atoms.

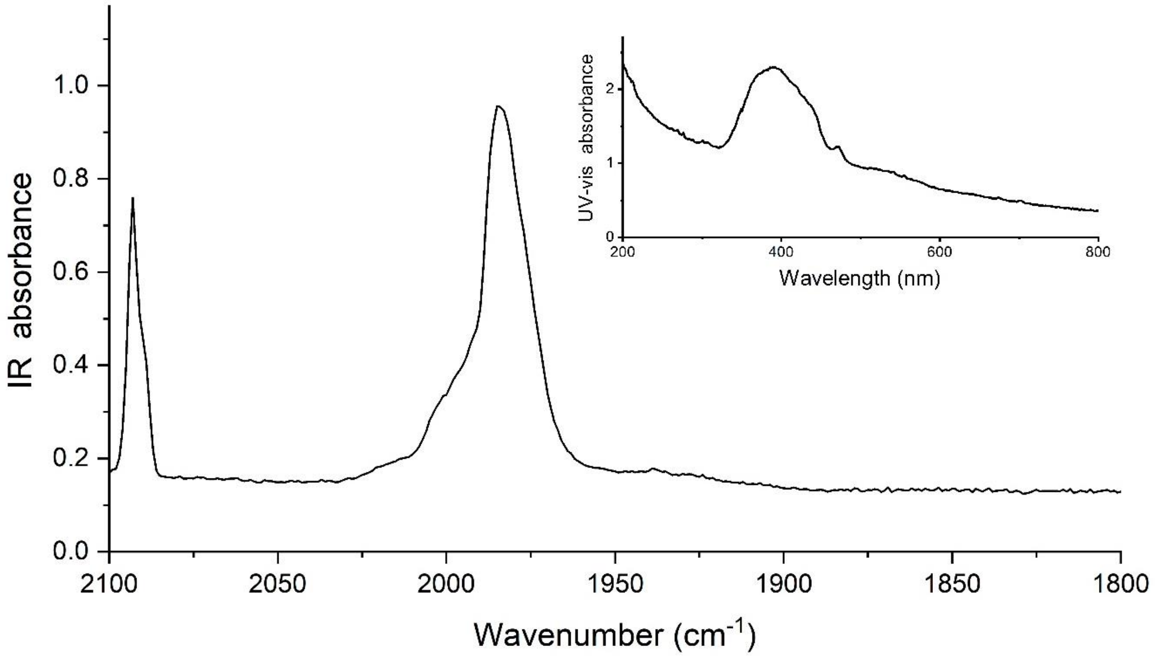

Figure S8. FT-IR spectrum of the cocondensation of dysprosium atoms with neat CO at 10 K with strong band centered at 1983 cm^{-1}.
Insert: UV-vis spectrum of the condensation of dysprosium atoms with neat CO at 10 K, with broad band centered at 387 nm overlappimg with weak spectral features of unreacted metal atoms.

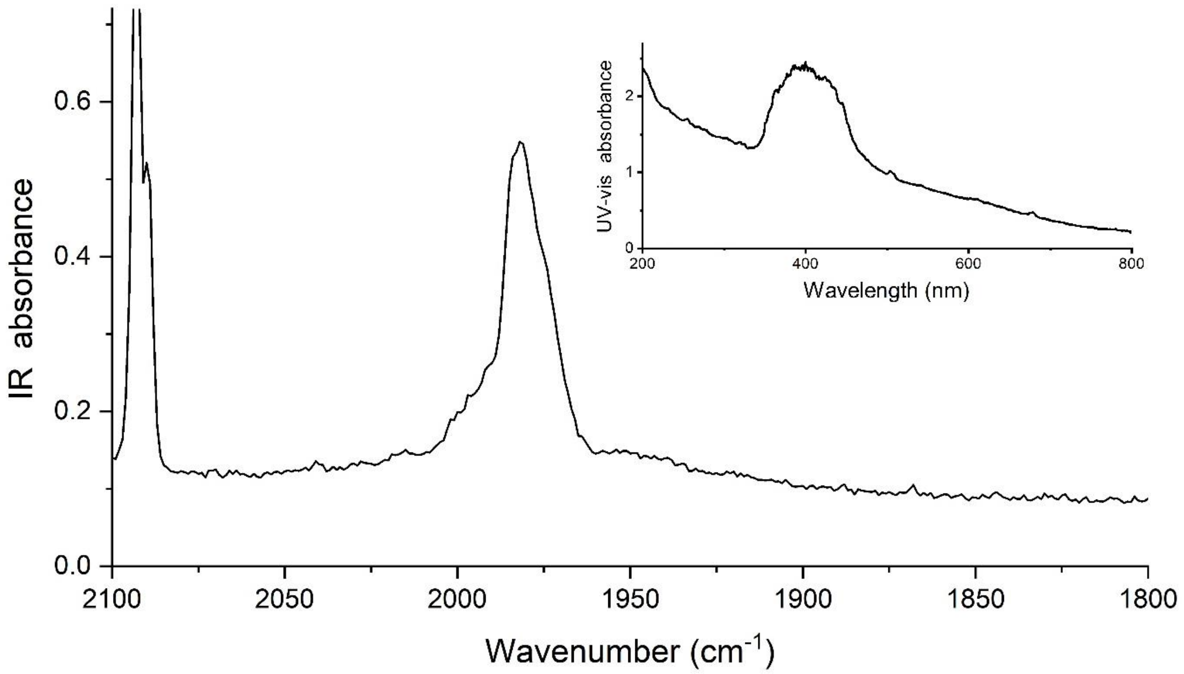

Figure S9. FT-IR spectrum of the cocondensation of holmium atoms with neat CO at 10 K with strong band centered at 1980 cm^{-1}.
Insert: UV-vis spectrum of the condensation of holmium atoms with neat CO at 10 K, with broad band centered at 387 nm.

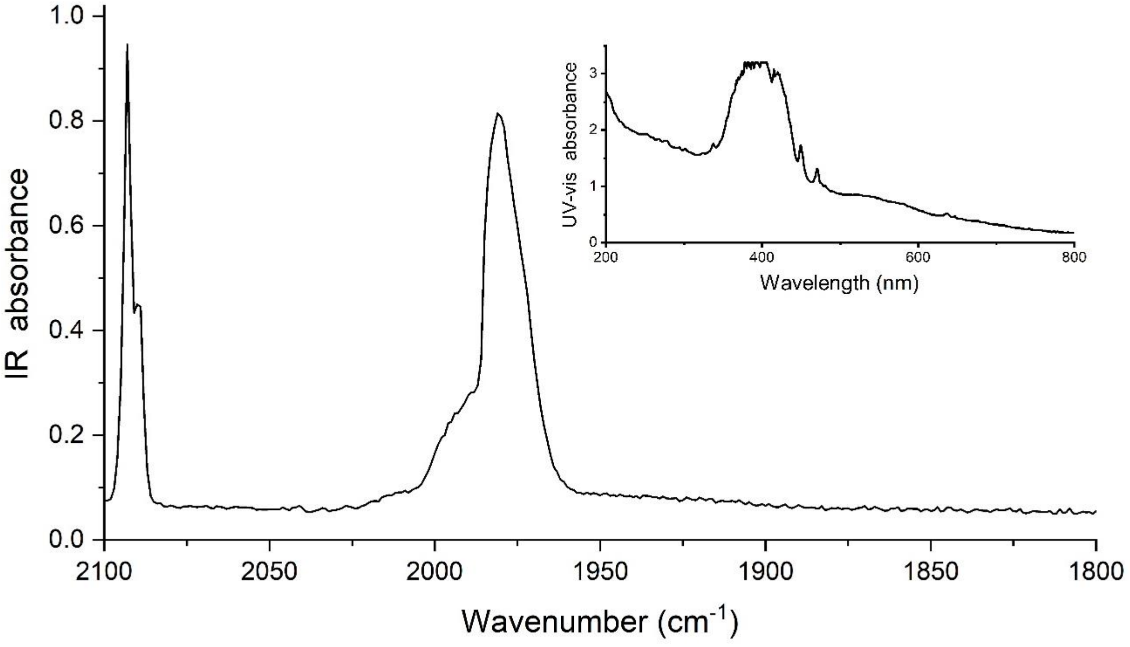

Figure S10. FT-IR spectrum of the cocondensation of erbium atoms with neat CO at 10 K with strong band centered at 1980 cm^{-1}.
Insert: UV-vis spectrum of the condensation of erbium atoms with neat CO at 10 K, with broad band centered at 388 nm overlapping with spectral features of unreacted metal atoms.

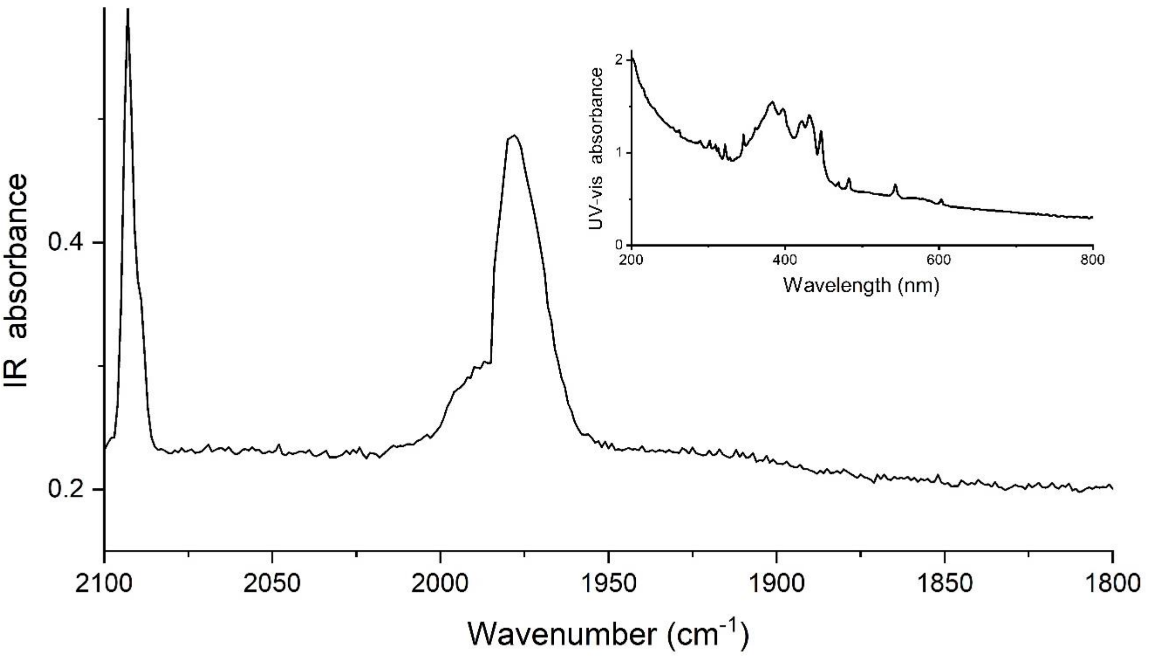

Figure S11. FT-IR spectrum of the cocondensation of thulium atoms with neat CO at 10 K with strong band centered at 1977 cm^{-1}.
Insert: UV-vis spectrum of the condensation of thulium atoms with neat CO at 10 K, with broad band centered at 382 nm overlapping with strong spectral features of unreacted metal atoms.

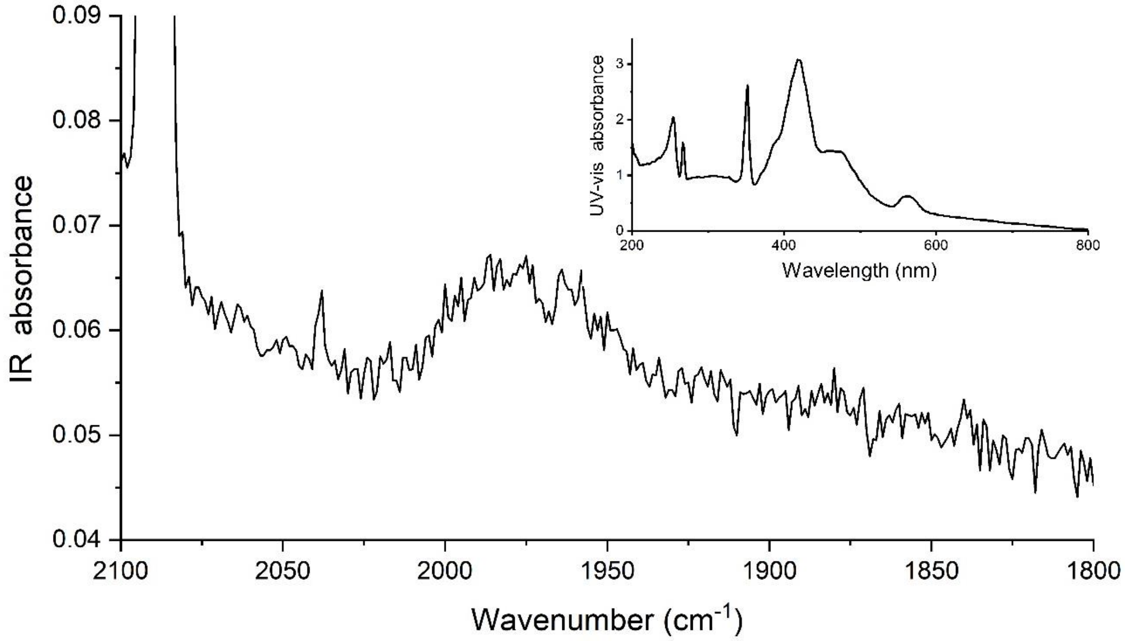

Figure S12. FT-IR spectrum of the cocondensation of ytterbium atoms with neat CO at 10 K with weak band centered at 1975 cm^{-1}.
Insert: UV-vis spectrum of the condensation of ytterbium atoms with neat CO at 10 K, with broad band centered at 368 nm overlapping with strong spectral features of unreacted metal atoms.

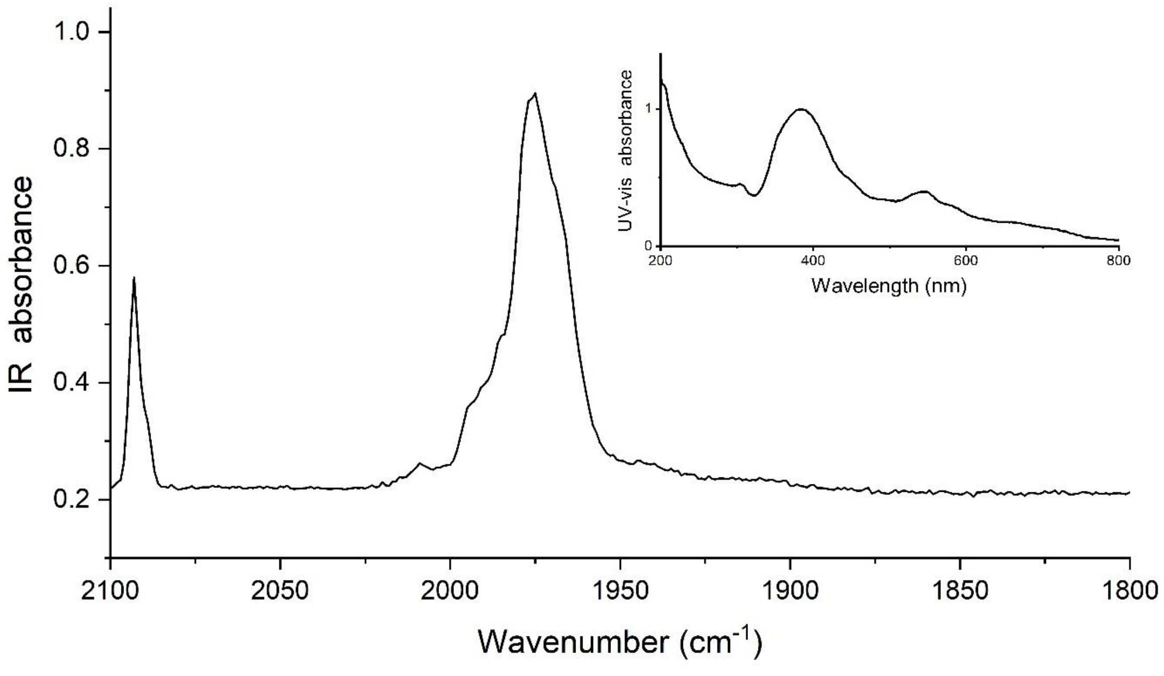

Figure S13. FT-IR spectrum of the cocondensation of lutetium atoms with neat CO at 10 K with strong band centered at 1974 cm^{-1}.
Insert: UV-vis spectrum of the condensation of lutetium atoms with neat CO at 10 K, with broad band centered at 385 nm and weak further features.

Cartesian coordinates of the optimized structures

$^{2}La(CO)_8$ BP86

La	0.00000000	0.00000000	0.00000000
C	0.00000000	2.23604900	1.45265000
C	0.00000000	-2.23604900	1.45265000
C	2.23604900	0.00000000	1.45265000
C	-2.23604900	0.00000000	1.45265000
C	0.00000000	-2.23604900	-1.45265000
C	0.00000000	2.23604900	-1.45265000
C	-2.23604900	0.00000000	-1.45265000
C	2.23604900	0.00000000	-1.45265000
O	0.00000000	-3.21470900	2.05578600
O	0.00000000	3.21470900	2.05578600
O	-3.21470900	0.00000000	2.05578600
O	3.21470900	0.00000000	2.05578600
O	0.00000000	3.21470900	-2.05578600
O	0.00000000	-3.21470900	-2.05578600
O	3.21470900	0.00000000	-2.05578600
O	-3.21470900	0.00000000	-2.05578600

$^{2}La(CO)_8$ B3LYP

La	0.00000000	0.00000000	0.00000000
C	0.00000000	2.24584000	1.50348600
C	0.00000000	-2.24584000	1.50348600
C	2.24584000	0.00000000	1.50348600
C	-2.24584000	0.00000000	1.50348600
C	0.00000000	-2.24584000	-1.50348600
C	0.00000000	2.24584000	-1.50348600
C	-2.24584000	0.00000000	-1.50348600
C	2.24584000	0.00000000	-1.50348600
O	0.00000000	-3.20097700	2.11826000
O	0.00000000	3.20097700	2.11826000
O	-3.20097700	0.00000000	2.11826000
O	3.20097700	0.00000000	2.11826000
O	0.00000000	3.20097700	-2.11826000
O	0.00000000	-3.20097700	-2.11826000
O	3.20097700	0.00000000	-2.11826000
O	-3.20097700	0.00000000	-2.11826000

$^{2}La(CO)_8^-$ BP86

La	0.00000000	0.00000000	0.00000000
C	0.00000000	2.14867000	1.52005800
C	0.00000000	-2.14867000	1.52005800
C	2.14867000	0.00000000	1.52005800
C	-2.14867000	0.00000000	1.52005800
C	0.00000000	-2.14867000	-1.52005800
C	0.00000000	2.14867000	-1.52005800
C	-2.14867000	0.00000000	-1.52005800
C	2.14867000	0.00000000	-1.52005800
O	0.00000000	-3.09648700	2.19080900
O	0.00000000	3.09648700	2.19080900
O	-3.09648700	0.00000000	2.19080900
O	3.09648700	0.00000000	2.19080900
O	0.00000000	3.09648700	-2.19080900
O	0.00000000	-3.09648700	-2.19080900
O	3.09648700	0.00000000	-2.19080900
O	-3.09648700	0.00000000	-2.19080900

$^{2}La(CO)_8Ar_8$ BP86

La	-0.00000000	-0.00000000	0.00000000
C	0.00000000	-2.23813861	1.44860732
C	-0.00000000	2.23813861	1.44860732
C	-2.23813861	-0.00000000	1.44860732
C	2.23813861	0.00000000	1.44860732
C	0.00000000	2.23813861	-1.44860732
C	-0.00000000	-2.23813861	-1.44860732
C	2.23813861	-0.00000000	-1.44860732
C	-2.23813861	0.00000000	-1.44860732
O	0.00000000	3.21930341	2.04743353
O	0.00000000	-3.21930341	2.04743353
O	3.21930341	0.00000000	2.04743353
O	-3.21930341	-0.00000000	2.04743353
O	-0.00000000	-3.21930341	-2.04743353
O	-0.00000000	3.21930341	-2.04743353
O	-3.21930341	0.00000000	-2.04743353
O	3.21930341	-0.00000000	-2.04743353
Ar	-6.40815387	0.00000000	-3.96961816
Ar	6.40815387	0.00000000	3.96961816
Ar	6.40815387	0.00000000	-3.96961816
Ar	-6.40815387	0.00000000	3.96961816
Ar	-0.00000000	6.40815387	-3.96961816
Ar	-0.00000000	-6.40815387	3.96961816
Ar	0.00000000	6.40815387	3.96961816
Ar	0.00000000	-6.40815387	-3.96961816

$^{2}La(CO)_8(CO)_8$ BP86

La	-0.00000000	-0.00000000	0.00000000
C	0.00000000	-2.24233081	1.44296509
C	-0.00000000	2.24233081	1.44296509
C	-2.24233081	-0.00000000	1.44296509
C	2.24233081	0.00000000	1.44296509
C	0.00000000	2.24233081	-1.44296509
C	-0.00000000	-2.24233081	-1.44296509
C	2.24233081	-0.00000000	-1.44296509
C	-2.24233081	0.00000000	-1.44296509
O	0.00000000	3.22387568	2.04092014
O	0.00000000	-3.22387568	2.04092014
O	3.22387568	0.00000000	2.04092014
O	-3.22387568	-0.00000000	2.04092014
O	-0.00000000	-3.22387568	-2.04092014
O	-0.00000000	3.22387568	-2.04092014
O	-3.22387568	0.00000000	-2.04092014
O	3.22387568	-0.00000000	-2.04092014
C	-7.41131516	0.00000000	4.50170891
C	7.41131516	0.00000000	-4.50170891
C	7.41131516	0.00000000	4.50170891
C	-0.00000000	7.41131516	4.50170891
C	-7.41131516	-0.00000000	-4.50170891
C	-0.00000000	-7.41131516	-4.50170891
C	0.00000000	-7.41131516	4.50170891
C	0.00000000	7.41131516	-4.50170891
O	8.43839034	-0.00000000	4.99519835
O	-0.00000000	8.43839034	4.99519835
O	8.43839034	-0.00000000	-4.99519835
O	-0.00000000	-8.43839034	-4.99519835
O	-8.43839034	-0.00000000	-4.99519835
O	-8.43839034	-0.00000000	4.99519835
O	0.00000000	8.43839034	-4.99519835
O	-0.00000000	-8.43839034	4.99519835

^{4}La(CO)$_{8}$ BP86

La	0.00000000	0.00000000	0.00000000
C	0.00000000	2.08821300	1.69135200
C	0.00000000	-2.08821300	1.69135200
C	2.08821300	0.00000000	1.69135200
C	-2.08821300	0.00000000	1.69135200
C	1.47659000	-1.47659000	-1.69135200
C	-1.47659000	1.47659000	-1.69135200
C	-1.47659000	-1.47659000	-1.69135200
C	1.47659000	1.47659000	-1.69135200
O	0.00000000	-2.99045800	2.40625000
O	0.00000000	2.99045800	2.40625000
O	-2.99045800	0.00000000	2.40625000
O	2.99045800	0.00000000	2.40625000
O	-2.11457300	2.11457300	-2.40625000
O	2.11457300	-2.11457300	-2.40625000
O	2.11457300	2.11457300	-2.40625000
O	-2.11457300	-2.11457300	-2.40625000

^{4}La(CO)$_{8}$ B3LYP

La	0.00000000	0.00000000	0.00000000
C	0.00000000	2.14875800	1.66651400
C	0.00000000	-2.14875800	1.66651400
C	2.14875800	0.00000000	1.66651400
C	-2.14875800	0.00000000	1.66651400
C	1.51940200	-1.51940200	-1.66651400
C	-1.51940200	1.51940200	-1.66651400
C	-1.51940200	-1.51940200	-1.66651400
C	1.51940200	1.51940200	-1.66651400
O	0.00000000	-3.07304400	2.32986700
O	0.00000000	3.07304400	2.32986700
O	-3.07304400	0.00000000	2.32986700
O	3.07304400	0.00000000	2.32986700
O	-2.17297000	2.17297000	-2.32986700
O	2.17297000	-2.17297000	-2.32986700
O	2.17297000	2.17297000	-2.32986700
O	-2.17297000	-2.17297000	-2.32986700

^{2}La(CO)$_{7}$ BP86

La	-0.00292600	0.06352600	0.29773700
C	-0.12192900	2.70668700	0.77565200
C	2.27876300	-0.82451000	1.38588500
C	2.16968000	1.09424100	-0.74705100
C	0.03711500	-0.52236400	-2.17330500
C	-2.22217700	-0.99064800	1.36570500
C	-2.23586600	0.93257900	-0.76715600
C	0.10126700	-2.48798700	-0.24271300
O	0.05785600	-0.81080200	-3.29103100
O	-3.23194800	1.30018800	-1.21560200
O	-3.21154500	-1.44164500	1.74080600
O	3.29447600	-1.20573000	1.76790000
O	3.13993200	1.53018000	-1.19086700
O	-0.17968700	3.84721800	0.90133400
O	0.14662700	-3.60302800	-0.53168200

^{4}La(CO)$_{7}$ BP86

La	0.00000000	0.00000000	0.09664000
C	1.57295400	0.90814500	-1.79601200
C	-1.57295400	0.90814500	-1.79601200
C	0.00000000	2.56793500	0.90774500
C	0.00000000	0.00000000	2.86574500
C	0.00000000	-1.81629000	-1.79601200
C	2.22389700	-1.28396700	0.90774500
C	-2.22389700	-1.28396700	0.90774500
O	0.00000000	0.00000000	4.01375700
O	3.20530300	-1.85058300	1.10144000
O	0.00000000	-2.50987000	-2.71911600
O	-2.17361100	1.25493500	-2.71911600
O	0.00000000	3.70116600	1.10144000
O	2.17361100	1.25493500	-2.71911600
O	-3.20530300	-1.85058300	1.10144000

^{2}La(CO)$_{7}$ B3LYP

La	-0.24642200	0.12791000	0.00000000
C	-2.52659500	-1.34494500	0.00000000
C	-0.39729500	1.63190300	-2.25383400
C	-0.09855600	-1.34686000	-2.21006300
C	2.00923100	-1.14529100	0.00000000
C	-0.39729500	1.63190300	2.25383400
C	-0.09855600	-1.34686000	2.21006300
C	1.87075000	1.72486800	0.00000000
O	3.00984000	-1.69248300	0.00000000
O	-0.02852700	-1.96074700	3.16595600
O	-0.39729500	2.21919800	3.22588300
O	-0.39729500	2.21919800	-3.22588300
O	-0.02852700	-1.96074700	-3.16595600
O	-3.46512700	-1.98415000	0.00000000
O	2.79142600	2.39483600	0.00000000

^{4}La(CO)$_{7}$ B3LYP

La	0.00000000	0.00000000	0.04585300
O	0.00000000	0.00000000	4.02357500
O	3.20125800	-1.84824700	1.14212600
O	0.00000000	-2.62537200	-2.66750700
O	-2.27363900	1.31268600	-2.66750700
O	0.00000000	3.69649400	1.14212600
O	2.27363900	1.31268600	-2.66750700
O	-3.20125800	-1.84824700	1.14212600
C	1.64356900	0.94891500	-1.78766200
C	-1.64356900	0.94891500	-1.78766200
C	0.00000000	2.58205800	0.92463700
C	0.00000000	0.00000000	2.89022500
C	0.00000000	-1.89783000	-1.78766200
C	2.23612800	-1.29102900	0.92463700
C	-2.23612800	-1.29102900	0.92463700

$^{2}La(CO)_{6}$ BP86

La	0.00000000	0.00000000	0.49000700
C	0.00000000	1.93556300	-1.12212900
C	-2.23158800	0.55661900	1.95107300
C	2.23158800	-0.55661900	1.95107300
C	-1.92046400	0.17623900	-1.16651000
C	1.92046400	-0.17623900	-1.16651000
C	0.00000000	-1.93556300	-1.12212900
O	-0.00375900	2.75987300	-1.93452100
O	-3.24083900	0.86567000	2.40572100
O	-2.75628800	0.24807600	-1.96367400
O	2.75628800	-0.24807600	-1.96367400
O	3.24083900	-0.86567000	2.40572100
O	0.00375900	-2.75987300	-1.93452100

$^{4}La(CO)_{6}$ BP86

La	0.00000000	0.00000000	0.00000000
C	0.00000000	0.00000000	2.65241400
C	0.00000000	2.65241400	0.00000000
C	-2.65241400	0.00000000	0.00000000
C	2.65241400	0.00000000	0.00000000
C	0.00000000	-2.65241400	0.00000000
C	0.00000000	0.00000000	-2.65241400
O	0.00000000	0.00000000	3.80413700
O	0.00000000	3.80413700	0.00000000
O	3.80413700	0.00000000	0.00000000
O	0.00000000	-3.80413700	0.00000000
O	-3.80413700	0.00000000	0.00000000
O	0.00000000	0.00000000	-3.80413700

$^{2}La(CO)_{6}$ B3LYP

La	0.00000000	0.00000000	0.35762100
C	0.00000000	2.07504700	-1.18623800
C	-1.97560400	0.66576500	2.11825700
C	1.97560400	-0.66576500	2.11825700
C	-2.05339300	0.15497000	-1.22935300
C	2.05339300	-0.15497000	-1.22935300
C	0.00000000	-2.07504700	-1.18623800
O	-0.01058600	2.94952300	-1.91947800
O	-2.81392600	1.00714100	2.80418600
O	-2.94787400	0.22304900	-1.93573200
O	2.94787400	-0.22304900	-1.93573200
O	2.81392600	-1.00714100	2.80418600
O	0.01058600	-2.94952300	-1.91947800

$^{4}La(CO)_{6}$ B3LYP

La	0.00000000	0.00000000	0.00000000
C	0.00000000	0.00000000	2.67677300
C	0.00000000	2.67677300	0.00000000
C	-2.67677300	0.00000000	0.00000000
C	2.67677300	0.00000000	0.00000000
C	0.00000000	-2.67677300	0.00000000
C	0.00000000	0.00000000	-2.67677300
O	0.00000000	0.00000000	3.81465900
O	0.00000000	3.81465900	0.00000000
O	3.81465900	0.00000000	0.00000000
O	0.00000000	-3.81465900	0.00000000
O	-3.81465900	0.00000000	0.00000000
O	0.00000000	0.00000000	-3.81465900

$^{3}Ce(CO)_{8}$ B3LYP

Ce	0.00000000	0.00000000	0.00000000
C	0.00000000	2.11647013	-1.59606072
C	-2.11647013	0.00000000	1.59606072
C	0.00000000	2.11647013	1.59606072
C	-2.11647013	-0.00000000	-1.59606072
C	2.11647013	-0.00000000	1.59606072
C	0.00000000	-2.11647013	-1.59606072
C	-0.00000000	-2.11647013	1.59606072
C	2.11647013	0.00000000	-1.59606072
O	-3.00748763	0.00000000	2.30092374
O	0.00000000	3.00748763	-2.30092374
O	-3.00748763	-0.00000000	-2.30092374
O	-0.00000000	3.00748763	2.30092374
O	-0.00000000	-3.00748763	-2.30092374
O	3.00748763	0.00000000	2.30092374
O	3.00748763	0.00000000	-2.30092374
O	0.00000000	-3.00748763	2.30092374

$^{5}Ce(CO)_{8}$ B3LYP

Ce	0.00000000	0.00000000	0.00011306
C	1.18227958	1.74927750	-1.63844112
C	1.17586358	-1.75523396	1.63612639
C	1.76015259	-1.20836657	-1.61797565
C	1.76886755	1.20022583	1.61537099
C	-1.18227958	-1.74927750	-1.63844112
C	-1.17586358	1.75523396	1.63612639
C	-1.76886755	-1.20022583	1.61537099
C	-1.76015259	1.20836657	-1.61797565
O	1.68025994	-2.50669229	2.32587454
O	1.68967619	2.49752594	-2.32946820
O	2.52323609	1.72216930	2.28784596
O	2.51059802	-1.73425726	-2.29179053
O	-1.68025994	2.50669229	2.32587454
O	-1.68967619	-2.49752594	-2.32946820
O	-2.51059802	1.73425726	-2.29179053
O	-2.52323609	-1.72216930	2.28784596

$^{4}Pr(CO)_{8}$ B3LYP

Pr	0.00000000	0.00000000	0.00000000
C	0.00000000	-2.18355419	1.46113103
C	-0.00000000	2.18355419	1.46113103
C	-2.18355419	-0.00000000	1.46113103
C	2.18355419	0.00000000	1.46113103
C	-2.18355419	0.00000000	-1.46113103
C	2.18355419	-0.00000000	-1.46113103
C	0.00000000	2.18355419	-1.46113103
C	-0.00000000	-2.18355419	-1.46113103
O	0.00000000	3.13814002	2.07727042
O	0.00000000	-3.13814002	2.07727042
O	3.13814002	0.00000000	2.07727042
O	-3.13814002	-0.00000000	2.07727042
O	3.13814002	-0.00000000	-2.07727042
O	-3.13814002	0.00000000	-2.07727042
O	-0.00000000	-3.13814002	-2.07727042
O	-0.00000000	3.13814002	-2.07727042

$^{6}Pr(CO)_8$ B3LYP

Pr	0.00000000	0.00000000	0.00000000
C	0.00000000	2.18985900	1.54925100
C	0.00000000	-2.18985900	1.54925100
C	2.18985900	0.00000000	1.54925100
C	-2.18985900	0.00000000	1.54925100
C	2.18985900	0.00000000	-1.54925100
C	-2.18985900	0.00000000	-1.54925100
C	0.00000000	-2.18985900	-1.54925100
C	0.00000000	2.18985900	-1.54925100
O	0.00000000	-3.11743100	2.20534200
O	0.00000000	3.11743100	2.20534200
O	-3.11743100	0.00000000	2.20534200
O	3.11743100	0.00000000	2.20534200
O	-3.11743100	0.00000000	-2.20534200
O	3.11743100	0.00000000	-2.20534200
O	0.00000000	3.11743100	-2.20534200
O	0.00000000	-3.11743100	-2.20534200

$^{4}Pr(CO)_7$ B3LYP

Pr	0.00002300	0.05764100	-0.25389500
O	-0.00116100	-3.62088900	0.37923900
O	-0.00038500	-0.81894800	3.29677600
O	3.06239200	1.45266800	1.32962900
O	3.19122800	-1.25912700	-1.79242400
O	-3.19186200	-1.25743100	-1.79247000
O	-3.06163100	1.45454800	1.32933900
O	0.00128500	3.70546600	-1.16512700
C	0.00088500	2.59882800	-0.90771000
C	-2.20893500	-0.86344600	-1.38264200
C	-2.12051900	1.03216000	0.84683800
C	-0.00026300	-0.54050000	2.19038700
C	2.20850700	-0.86464900	-1.38257300
C	2.12106800	1.03087200	0.84702600
C	-0.00079000	-2.50178300	0.17202800

$^{6}Pr(CO)_7$ B3LYP

Pr	0.00003800	-0.00002800	0.27950600
O	3.49321700	-0.88225500	-0.87381700
O	0.00002200	0.00000300	-3.48530900
O	-2.51121700	-2.58354600	-0.87356600
O	0.98616300	-3.48651500	1.46288200
O	2.52623300	2.59719500	1.46302100
O	-0.98249900	3.46624600	-0.87374100
O	-3.51233600	0.88919900	1.46272800
C	-2.44683100	0.62177800	1.16768400
C	1.76194400	1.80815300	1.16783100
C	-0.68262700	2.42002600	-0.53741100
C	0.00005300	-0.00003200	-2.34935200
C	0.68500500	-2.43003600	1.16789200
C	-1.75461200	-1.80118100	-0.53731300
C	2.43724900	-0.61887100	-0.53740600

$^{4}Pr(CO)_6$ B3LYP

Pr	0.00000000	0.00000000	0.31535900
C	0.00000000	2.04993100	-1.13499000
C	-1.84472700	0.84737000	2.06115000
C	1.84472700	-0.84737000	2.06115000
C	-2.02263400	0.15832900	-1.18938300
C	2.02263400	-0.15832900	-1.18938300
C	0.00000000	-2.04993100	-1.13499000
O	-0.01307500	2.95088400	-1.83644900
O	-2.64531300	1.27298600	2.74413600
O	-2.93473100	0.23238600	-1.87315500
O	2.93473100	-0.23238600	-1.87315500
O	2.64531300	-1.27298600	2.74413600
O	0.01307500	-2.95088400	-1.83644900

$^{6}Pr(CO)_6$ B3LYP

Pr	0.00000000	0.00000000	0.00000000
C	-0.00000100	0.00000100	2.60083300
C	-2.35287100	1.12152700	0.00000000
C	-1.12152700	-2.35287000	0.00000000
C	1.12152700	2.35287000	0.00000000
C	2.35287100	-1.12152600	0.00000000
C	0.00000100	-0.00000100	-2.60083300
O	0.00000100	-0.00000100	3.73899600
O	-3.38011700	1.61117900	0.00000000
O	1.61117800	3.38011700	0.00000000
O	3.38011700	-1.61117800	0.00000000
O	-1.61117900	-3.38011700	0.00000000
O	-0.00000100	0.00000000	-3.73899600

$^{5}Nd(CO)_8$ B3LYP

Nd	0.00000000	0.00000000	0.00000000
C	0.00000000	2.16082300	1.45690400
C	0.00000000	-2.16082300	1.45690400
C	2.16082300	0.00000000	1.45690400
C	-2.16082300	0.00000000	1.45690400
C	2.16082300	0.00000000	-1.45690400
C	-2.16082300	0.00000000	-1.45690400
C	0.00000000	-2.16082300	-1.45690400
C	0.00000000	2.16082300	-1.45690400
O	0.00000000	-3.11301700	2.07697400
O	0.00000000	3.11301700	2.07697400
O	-3.11301700	0.00000000	2.07697400
O	3.11301700	0.00000000	2.07697400
O	-3.11301700	0.00000000	-2.07697400
O	3.11301700	0.00000000	-2.07697400
O	0.00000000	3.11301700	-2.07697400
O	0.00000000	-3.11301700	-2.07697400

$^{7}Nd(CO)_8$ B3LYP

Nd	-0.00000000	0.00000000	0.00000000
C	-2.72643289	0.00788803	0.00000000
C	0.76228189	-2.55617492	0.00000000
C	-0.77230082	-1.27571599	2.21111993
C	-0.77230082	-1.27571599	-2.21111993
C	0.77230082	1.27571599	2.21111993
C	0.77230082	1.27571599	-2.21111993
C	2.72643289	-0.00788803	0.00000000
C	-0.76228189	2.55617492	0.00000000
O	1.05758622	-3.65443784	0.00000000
O	-3.85868589	0.01157169	0.00000000
O	-1.07111286	-1.81861534	-3.16479085
O	-1.07111286	-1.81861534	3.16479085
O	1.07111286	1.81861534	-3.16479085
O	1.07111286	1.81861534	3.16479085
O	-1.05758622	3.65443784	0.00000000
O	3.85868589	-0.01157169	0.00000000

$^{5}Nd(CO)_7$ B3LYP

Nd	-0.00263100	-0.05708300	-0.22893700
C	-0.12253700	-2.57612100	-0.92181200
C	2.19783800	0.74656800	-1.39763500
C	2.07147600	-1.10612300	0.86132800
C	0.04315300	0.57215800	2.19266100
C	-2.14623500	0.91336400	-1.37668700
C	-2.14554000	-0.93116000	0.88502500
C	0.10789800	2.47927000	0.12203600
O	0.16104100	3.60425400	0.29073100
O	0.06518400	0.86709000	3.29451600
O	-3.10463500	-1.30615000	1.37064500
O	-3.11037900	1.33048300	-1.80854600
O	3.18241800	1.10048500	-1.83977000
O	2.99575000	-1.56669100	1.34097200
O	-0.17418900	-3.67481500	-1.20520800

$^{7}Nd(CO)_7$ B3LYP

Nd	-0.00000000	-0.00000000	-0.28875104
C	2.19967254	1.26998153	-1.14774321
C	-2.19967254	1.26998153	-1.14774321
C	0.00000000	2.51374076	0.52569243
C	0.00000000	0.00000000	2.35444596
C	0.00000000	-2.53996306	-1.14774321
C	2.17696335	-1.25687038	0.52569243
C	-2.17696335	-1.25687038	0.52569243
O	-3.11860555	-1.80052775	0.86407546
O	-0.00000000	0.00000000	3.49007596
O	3.11860555	-1.80052775	0.86407546
O	0.00000000	-3.64167882	-1.42766512
O	-3.15378637	1.82083941	-1.42766512
O	0.00000000	3.60105550	0.86407546
O	3.15378637	1.82083941	-1.42766512

$^{5}Nd(CO)_6$ B3LYP

Nd	0.50640988	-0.01878825	0.00000000
C	0.54903188	1.99260689	1.59421904
C	0.49464098	-2.02507812	1.61150088
C	0.49464098	-2.02507812	-1.61150088
C	-1.52656808	0.04774095	1.38893906
C	0.54903188	1.99260689	-1.59421904
C	-1.52656808	0.04774095	-1.38893906
O	0.49898688	2.88662051	2.29779252
O	0.42479302	-2.90089951	2.33546341
O	-2.46387006	0.07495158	2.04376611
O	0.49898688	2.88662051	-2.29779252
O	0.42479302	-2.90089951	-2.33546341
O	-2.46387006	0.07495158	-2.04376611

$^{7}Nd(CO)_6$ B3LYP

Nd	0.00000000	0.00000000	0.00000000
C	0.00000000	0.00000000	2.57945600
C	0.00000000	-2.57945600	-0.00000000
C	2.57945600	0.00000000	-0.00000000
C	-2.57945600	0.00000000	0.00000000
C	0.00000000	2.57945600	0.00000000
C	-0.00000000	0.00000000	-2.57945600
O	0.00000000	0.00000000	3.71749100
O	0.00000000	-3.71749100	-0.00000000
O	-3.71749100	0.00000000	0.00000000
O	0.00000000	3.71749100	0.00000000
O	3.71749100	0.00000000	-0.00000000
O	-0.00000000	0.00000000	-3.71749100

$^{7}Sm(CO)_8$ B3LYP

Sm	-0.05268200	-0.00057200	-0.00003600
C	0.11702000	2.13348500	1.50987200
C	-0.03031300	-2.18062800	1.46399500
C	2.13021000	-0.07383400	1.42430100
C	-2.19817200	0.04369400	1.54978400
C	2.12756200	0.10690800	-1.42620700
C	-2.19814800	-0.07729000	-1.54865400
C	0.14890800	-2.13202000	-1.50982100
C	-0.06524100	2.18000100	-1.46324200
O	-0.01899800	-3.12938500	2.08801300
O	0.22315600	3.06179700	2.15611700
O	-3.10695900	0.06040400	2.22945200
O	3.09897900	-0.09883200	2.01839500
O	-3.10688400	-0.10748200	-2.22792400
O	3.09529400	0.14722900	-2.02114800
O	-0.06908300	3.12918500	-2.08670900
O	0.26891300	-3.05872400	-2.15594000

$^{9}Sm(CO)_8$ B3LYP

Sm	0.00000000	0.00000000	0.00000000
C	0.00000000	2.19768400	1.55417400
C	0.00000000	-2.19768400	1.55417400
C	2.19768400	0.00000000	1.55417400
C	-2.19768400	0.00000000	1.55417400
C	2.19768400	0.00000000	-1.55417400
C	-2.19768400	0.00000000	-1.55417400
C	0.00000000	-2.19768400	-1.55417400
C	0.00000000	2.19768400	-1.55417400
O	0.00000000	-3.12411800	2.20963700
O	0.00000000	3.12411800	2.20963700
O	-3.12411800	0.00000000	2.20963700
O	3.12411800	0.00000000	2.20963700
O	-3.12411800	0.00000000	-2.20963700
O	3.12411800	0.00000000	-2.20963700
O	0.00000000	3.12411800	-2.20963700
O	0.00000000	-3.12411800	-2.20963700

$^{7}Sm(CO)_7$ B3LYP

Sm	-0.06785500	0.02065700	-0.08347300
C	1.50354100	-1.72935000	1.06256000
C	-2.01774400	-1.38716400	-1.20715000
C	1.42697300	-1.17278100	-1.86455900
C	1.43137700	1.88771000	-1.13199100
C	-1.67210100	-0.47021800	1.94909900
C	1.50792900	1.05776900	1.73015500
C	-2.01460300	1.78974800	-0.44131700
O	-2.90006500	2.49012100	-0.56324100
O	2.11045700	2.70061400	-1.54596500
O	2.19435300	1.48162800	2.53171400
O	-2.39057900	-0.67663300	2.80415200
O	-2.90427600	-1.95334200	-1.63489500
O	2.10413400	-1.71173900	-2.60235500
O	2.18782300	-2.47252400	1.58490500

$^{9}Sm(CO)_7$ B3LYP

Sm	0.00000000	0.00000000	-0.19165915
O	0.00000000	3.59837797	0.99749313
O	0.00000000	0.00000000	3.57062185
O	-3.11628673	-1.79918898	0.99749313
O	-3.00943238	1.73749660	-1.78336450
O	3.00943238	1.73749660	-1.78336450
O	3.11628673	-1.79918898	0.99749313
O	0.00000000	-3.47499319	-1.78336450
C	0.00000000	-2.43032816	-1.34117501
C	2.10472593	1.21516408	-1.34117501
C	2.17889089	-1.25798324	0.65106512
C	0.00000000	0.00000000	2.43346385
C	-2.10472593	1.21516408	-1.34117501
C	-2.17889089	-1.25798324	0.65106512
C	-0.00000000	2.51596648	0.65106512

$^{7}Sm(CO)_6$ B3LYP

Sm	0.00000000	-0.00000000	-0.03000797
C	-1.84153210	-1.65395175	-0.90784596
C	-1.84153210	1.65395175	-0.90784596
C	1.84153210	1.65395175	-0.90784596
C	-1.86938410	0.00000000	1.83887500
C	1.84153210	-1.65395175	-0.90784596
C	1.86938410	0.00000000	1.83887500
O	-2.69310611	-2.32900474	-1.24527996
O	-2.69310611	2.32900474	-1.24527996
O	-2.73208411	0.00000000	2.57956298
O	2.69310611	-2.32900474	-1.24527996
O	2.69310611	2.32900474	-1.24527996
O	2.73208411	0.00000000	2.57956298

$^{9}Sm(CO)_6$ B3LYP

Sm	0.00000000	0.00000000	-0.46360381
C	-1.57101200	-2.13500598	-0.58862682
C	-1.57101200	2.13500598	-0.58862682
C	1.57101200	2.13500598	-0.58862682
C	-1.48038300	0.00000000	1.66492719
C	1.57101200	-2.13500598	-0.58862682
C	1.48038300	0.00000000	1.66492719
O	-2.22484200	-3.06559298	-0.58129282
O	-2.22484200	3.06559298	-0.58129282
O	-2.13986900	0.00000000	2.59428119
O	2.22484200	-3.06559298	-0.58129282
O	2.22484200	3.06559298	-0.58129282
O	2.13986900	0.00000000	2.59428119

$^{10}Eu(CO)_8$ BP86

Eu	0.00000000	0.00000000	0.00000000
C	1.51435000	1.51435000	1.51435000
C	-1.51435000	1.51435000	-1.51435000
C	-1.51435000	1.51435000	1.51435000
C	1.51435000	1.51435000	-1.51435000
C	-1.51435000	-1.51435000	-1.51435000
C	1.51435000	-1.51435000	1.51435000
C	1.51435000	-1.51435000	-1.51435000
C	-1.51435000	-1.51435000	1.51435000
O	-2.17753100	2.17753100	-2.17753100
O	2.17753100	2.17753100	2.17753100
O	2.17753100	2.17753100	-2.17753100
O	-2.17753100	2.17753100	2.17753100
O	2.17753100	-2.17753100	2.17753100
O	-2.17753100	-2.17753100	-2.17753100
O	-2.17753100	-2.17753100	2.17753100
O	2.17753100	-2.17753100	-2.17753100

$^{8}Eu(CO)_8$ BP86

Eu	0.00000000	0.00000000	0.00000000
C	0.00000000	2.05026400	1.54974500
C	1.44975600	1.44975600	-1.54974500
C	-1.44975600	1.44975600	-1.54974500
C	2.05026400	0.00000000	1.54974500
C	1.44975600	-1.44975600	-1.54974500
C	-2.05026400	0.00000000	1.54974500
C	0.00000000	-2.05026400	1.54974500
C	-1.44975600	-1.44975600	-1.54974500
O	2.07644200	2.07644200	-2.28060400
O	0.00000000	2.93653300	2.28060400
O	2.93653300	0.00000000	2.28060400
O	-2.07644200	2.07644200	-2.28060400
O	-2.93653300	0.00000000	2.28060400
O	2.07644200	-2.07644200	-2.28060400
O	-2.07644200	-2.07644200	-2.28060400
O	0.00000000	-2.93653300	2.28060400

$^{10}Eu(CO)_8$ B3LYP

Eu	0.00000000	0.00000000	0.00000000
C	1.56129400	1.56129400	1.56129400
C	-1.56129400	1.56129400	-1.56129400
C	-1.56129400	1.56129400	1.56129400
C	1.56129400	1.56129400	-1.56129400
C	-1.56129400	-1.56129400	-1.56129400
C	1.56129400	-1.56129400	1.56129400
C	1.56129400	-1.56129400	-1.56129400
C	-1.56129400	-1.56129400	1.56129400
O	-2.21648900	2.21648900	-2.21648900
O	2.21648900	2.21648900	2.21648900
O	2.21648900	2.21648900	-2.21648900
O	-2.21648900	2.21648900	2.21648900
O	2.21648900	-2.21648900	2.21648900
O	-2.21648900	-2.21648900	-2.21648900
O	-2.21648900	-2.21648900	2.21648900
O	2.21648900	-2.21648900	-2.21648900

$^{8}Eu(CO)_8$ B3LYP

Eu	0.00000000	0.00000000	0.00000000
C	0.00000000	2.13668700	1.60610900
C	1.51086600	1.51086600	-1.60610900
C	-1.51086600	1.51086600	-1.60610900
C	2.13668700	0.00000000	1.60610900
C	1.51086600	-1.51086600	-1.60610900
C	-2.13668700	0.00000000	1.60610900
C	0.00000000	-2.13668700	1.60610900
C	-1.51086600	-1.51086600	-1.60610900
O	2.13497000	2.13497000	-2.31924000
O	0.00000000	3.01930300	2.31924000
O	3.01930300	0.00000000	2.31924000
O	-2.13497000	2.13497000	-2.31924000
O	-3.01930300	0.00000000	2.31924000
O	2.13497000	-2.13497000	-2.31924000
O	-2.13497000	-2.13497000	-2.31924000
O	0.00000000	-3.01930300	2.31924000

$^{10}Eu(CO)_7$ BP86

Eu	0.00000000	0.00000000	-0.09981200
O	0.00000000	0.00000000	3.59720700
O	3.09493000	-1.78685900	0.96138600
O	0.00000000	-3.27391300	-1.93548300
O	-2.83529200	1.63695700	-1.93548300
O	0.00000000	3.57371700	0.96138600
O	2.83529200	1.63695700	-1.93548300
O	-3.09493000	-1.78685900	0.96138600
C	1.94851600	1.12497600	-1.41536600
C	-1.94851600	1.12497600	-1.41536600
C	0.00000000	2.46641000	0.64926100
C	0.00000000	0.00000000	2.44645000
C	0.00000000	-2.24995200	-1.41536600
C	2.13597300	-1.23320500	0.64926100
C	-2.13597300	-1.23320500	0.64926100

$^{8}Eu(CO)_7$ BP86

Eu	-0.35051690	0.02978705	0.00000000
C	1.17648871	2.07548034	0.00000000
C	1.59317260	-0.86614023	1.35215680
C	1.59317260	-0.86614023	-1.35215680
C	-0.96746294	-1.86106949	-1.62649131
C	-0.91777374	1.66195044	1.90026076
C	-0.91777374	1.66195044	-1.90026076
C	-0.96746294	-1.86106949	1.62649131
O	-1.12908996	-2.73591271	-2.35557004
O	-1.09684103	2.42027319	-2.74673039
O	-1.09684103	2.42027319	2.74673039
O	2.47732383	-1.29777091	1.95088470
O	2.47732383	-1.29777091	-1.95088470
O	1.81383153	3.03273146	0.00000000
O	-1.12908996	-2.73591271	2.35557004

$^{10}Eu(CO)_7$ B3LYP

Eu	0.00000000	0.00000000	-0.09957000
C	2.00822800	1.15945100	-1.44881900
C	-2.00822800	1.15945100	-1.44881900
C	0.00000000	2.54006600	0.67271500
C	0.00000000	0.00000000	2.53394400
C	0.00000000	-2.31890200	-1.44881900
C	2.19976200	-1.27003300	0.67271500
C	-2.19976200	-1.27003300	0.67271500
O	0.00000000	0.00000000	3.67078600
O	3.14911400	-1.81814200	0.97364600
O	0.00000000	-3.31764800	-1.98727900
O	-2.87316700	1.65882400	-1.98727900
O	0.00000000	3.63628400	0.97364600
O	2.87316700	1.65882400	-1.98727900
O	-3.14911400	-1.81814200	0.97364600

$^{8}Eu(CO)_7$ B3LYP

Eu	-0.03492600	0.03320200	-0.04394300
C	-1.60473100	-1.67683100	-1.38535300
C	2.00859500	0.00124900	-1.79371400
C	1.23413300	-2.28451000	0.18938000
C	0.67239700	-0.25265100	2.48195400
C	-1.42784500	2.03563500	-1.16635500
C	-2.35267300	0.02008500	1.27006400
C	1.55749300	2.10579300	0.51619800
O	0.97439000	-0.38412700	3.57041700
O	-3.36125800	0.01131200	1.79431500
O	-2.03930300	2.87474800	-1.62358200
O	2.88731700	-0.02869800	-2.51162900
O	1.78050800	-3.27980700	0.25689800
O	-2.27677300	-2.40504100	-1.93944200
O	2.24463400	2.98857300	0.71494400

$^{10}Eu(CO)_6$ BP86

Eu	0.00000000	0.00000000	0.30712100
C	0.00000000	2.49840800	0.96000500
C	2.16368500	-1.24920400	0.96000500
C	1.83979900	1.06220800	-1.13962800
C	-2.16368500	-1.24920400	0.96000500
C	-1.83979900	1.06220800	-1.13962800
C	0.00000000	-2.12441600	-1.13962800
O	0.00000000	3.64466100	1.07873500
O	3.15636900	-1.82233000	1.07873500
O	-3.15636900	-1.82233000	1.07873500
O	-2.68469600	1.55001000	-1.75020900
O	2.68469600	1.55001000	-1.75020900
O	0.00000000	-3.10002000	-1.75020900

$^{8}Eu(CO)_6$ BP86

Eu	0.52604000	-0.05777000	0.00000000
C	0.79394800	-1.16204000	2.32169200
C	0.79394800	-1.16204000	-2.32169200
C	0.20970400	1.76066900	-1.77933700
C	-1.17073000	-1.89602800	0.00000000
C	0.20970400	1.76066900	1.77933700
C	-1.81441400	0.76362100	0.00000000
O	0.79394800	-1.59874400	3.38749500
O	0.79394800	-1.59874400	-3.38749500
O	-1.98509200	-2.71433100	0.00000000
O	-0.05705500	2.57575800	2.54857000
O	-0.05705500	2.57575800	-2.54857000
O	-2.89788400	1.16660000	0.00000000

$^{10}Eu(CO)_6$ B3LYP

Eu	-0.00000000	0.00000000	0.00000000
C	0.00000000	2.41414392	1.09561870
C	2.09070996	-1.20707196	1.09561870
C	2.09070996	1.20707196	-1.09561870
C	-2.09070996	-1.20707196	1.09561870
C	-2.09070996	1.20707196	-1.09561870
C	-0.00000000	-2.41414392	-1.09561870
O	0.00000000	3.48536588	1.47805856
O	3.01841539	-1.74268294	1.47805856
O	-3.01841539	-1.74268294	1.47805856
O	-3.01841539	1.74268294	-1.47805856
O	3.01841539	1.74268294	-1.47805856
O	-0.00000000	-3.48536588	-1.47805856

$^{8}Eu(CO)_6$ B3LYP

Eu	0.00000000	0.00000000	0.43961600
C	2.13143300	1.59360000	0.61888800
C	-2.13143300	1.59360000	0.61888800
C	-2.13143300	-1.59360000	0.61888800
C	0.00000000	1.47278000	-1.69587600
C	2.13143300	-1.59360000	0.61888800
C	0.00000000	-1.47278000	-1.69587600
O	3.05507200	2.25692800	0.62099400
O	-3.05507200	2.25692800	0.62099400
O	0.00000000	2.12770500	-2.62940100
O	3.05507200	-2.25692800	0.62099400
O	-3.05507200	-2.25692800	0.62099400
O	0.00000000	-2.12770500	-2.62940100

$^{9}Gd(CO)_8$ BP86

Gd	0.00000000	0.00000000	0.00000000
C	0.00000000	2.00087200	1.61468400
C	-2.00087200	0.00000000	-1.61468400
C	-2.00087200	0.00000000	1.61468400
C	0.00000000	2.00087200	-1.61468400
C	0.00000000	-2.00087200	-1.61468400
C	2.00087200	0.00000000	1.61468400
C	2.00087200	0.00000000	-1.61468400
C	0.00000000	-2.00087200	1.61468400
O	-2.96459500	0.00000000	-2.24106600
O	0.00000000	2.96459500	2.24106600
O	0.00000000	2.96459500	-2.24106600
O	-2.96459500	0.00000000	2.24106600
O	2.96459500	0.00000000	2.24106600
O	0.00000000	-2.96459500	-2.24106600
O	0.00000000	-2.96459500	2.24106600
O	2.96459500	0.00000000	-2.24106600

$^{11}Gd(CO)_8$ BP86

Gd	0.00000600	-0.03206900	0.00045800
C	-1.85205300	-0.78542400	1.54249500
C	-0.81991700	-1.88118800	-1.40471400
C	0.91054000	-1.82809400	1.42200300
C	-2.04065300	0.76086900	-1.40765800
C	1.88943600	-0.71582600	-1.53684600
C	-0.78916100	1.96684100	1.53205300
C	0.69677500	1.98664500	-1.55130200
C	2.00488200	0.86849900	1.40154100
O	-1.18977400	-2.81075100	-1.98983300
O	-2.70685300	-1.24529000	2.16740400
O	-3.03169000	1.00529700	-1.93879300
O	1.32158500	-2.73502800	2.01476400
O	-1.21630100	2.85448700	2.12279800
O	2.76342200	-1.14506000	-2.15644600
O	2.98337700	1.16413900	1.92954900
O	1.07629800	2.88951700	-2.15128600

$^{9}Gd(CO)_8$ B3LYP

Gd	0.00000000	0.00000000	0.00000000
C	0.00000000	2.04921800	1.61071300
C	-2.04921800	0.00000000	-1.61071300
C	-2.04921800	0.00000000	1.61071300
C	0.00000000	2.04921800	-1.61071300
C	0.00000000	-2.04921800	-1.61071300
C	2.04921800	0.00000000	1.61071300
C	2.04921800	0.00000000	-1.61071300
C	0.00000000	-2.04921800	1.61071300
O	-3.01771900	0.00000000	-2.20418100
O	0.00000000	3.01771900	2.20418100
O	0.00000000	3.01771900	-2.20418100
O	-3.01771900	0.00000000	2.20418100
O	3.01771900	0.00000000	2.20418100
O	0.00000000	-3.01771900	-2.20418100
O	0.00000000	-3.01771900	2.20418100
O	3.01771900	0.00000000	-2.20418100

$^{11}Gd(CO)_8$ B3LYP

Gd	-0.00011100	-0.05708800	-0.00023300
C	-1.93715400	-0.80010700	1.45361500
C	-0.73253700	-1.87734700	-1.45963000
C	0.82414500	-1.84844200	1.45141200
C	-2.00312100	0.79824100	-1.52254400
C	1.97755900	-0.70210200	-1.45939000
C	-0.90368000	2.03863600	1.52607800
C	0.80721900	2.08890700	-1.51669400
C	1.96856400	0.88501100	1.52869000
O	-1.04648000	-2.78321000	-2.08861300
O	-2.78620100	-1.28713700	2.04011400
O	-2.96950900	0.98676700	-2.08926800
O	1.17705000	-2.74336900	2.07493500
O	-1.40074400	2.90066300	2.06689300
O	2.84715200	-1.15083300	-2.04559700
O	2.92570100	1.11559700	2.09501900
O	1.25317100	2.98112800	-2.05277000

$^{9}Gd(CO)_7$ BP86

Gd	-0.12786700	-0.05446600	0.00000000
C	-0.99343500	-1.82593500	-1.66621800
C	-1.70673400	1.94127900	0.00000000
C	0.23593200	1.49090400	-1.95177100
C	2.10087200	1.03151300	0.00000000
C	-0.99343500	-1.82593500	1.66621800
C	1.32483900	-2.14107300	0.00000000
C	0.23593200	1.49090400	1.95177100
O	3.18554900	1.42363300	0.00000000
O	2.07210000	-3.01665300	0.00000000
O	-1.32390100	-2.56065000	2.48356200
O	-2.46961900	2.80337100	0.00000000
O	0.36486500	2.11271700	-2.91339400
O	-1.32390100	-2.56065000	-2.48356200
O	0.36486500	2.11271700	2.91339400

$^{11}Gd(CO)_7$ BP86

Gd	0.00050993	0.04098909	0.00000000
C	1.78570776	-0.37749906	1.69682229
C	-0.00438717	-2.32525991	0.00000000
C	1.78570776	-0.37749906	-1.69682229
C	0.00278516	2.17307416	-1.64980175
C	-1.78990724	-0.36976892	1.69282597
C	0.00278516	2.17307416	1.64980175
C	-1.78990724	-0.36976892	-1.69282597
O	0.00206327	2.85836120	-2.56864272
O	0.00206327	2.85836120	2.56864272
O	-2.55459032	-0.73107392	2.47742089
O	-0.00723021	-3.49217491	0.00000000
O	2.55233612	-0.73861290	-2.47867866
O	2.55233612	-0.73861290	2.47867866
O	-2.55459032	-0.73107392	-2.47742089

$^{9}Gd(CO)_7$ B3LYP

Gd	-0.08368700	-0.04842200	0.00000000
C	-1.03276800	-1.81773700	-1.66847400
C	-1.79364200	1.87775400	0.00000000
C	0.21245500	1.48518300	-2.01983600
C	2.17307200	1.08864600	0.00000000
C	-1.03276800	-1.81773700	1.66847400
C	1.44255600	-2.12118500	0.00000000
C	0.21245500	1.48518300	2.01983600
O	3.24611300	1.47106700	0.00000000
O	2.20723900	-2.96276600	0.00000000
O	-1.43979000	-2.51658700	2.46355400
O	-2.62670100	2.65231200	0.00000000
O	0.29320100	2.06242700	-2.99780200
O	-1.43979000	-2.51658700	-2.46355400
O	0.29320100	2.06242700	2.99780200

$^{11}Gd(CO)_7$ B3LYP

Gd	0.00000000	0.00000000	-0.00535700
C	1.60747500	0.92807600	-1.58723700
C	-1.60747500	0.92807600	-1.58723700
C	0.00000000	2.47164300	0.82186600
C	0.00000000	0.00000000	2.81674400
C	0.00000000	-1.85615300	-1.58723700
C	2.14050600	-1.23582200	0.82186600
C	-2.14050600	-1.23582200	0.82186600
O	0.00000000	0.00000000	3.94720100
O	3.11165100	-1.79651300	0.99690900
O	0.00000000	-2.63434000	-2.42851600
O	-2.28140500	1.31717000	-2.42851600
O	0.00000000	3.59302500	0.99690900
O	2.28140500	1.31717000	-2.42851600
O	-3.11165100	-1.79651300	0.99690900

$^{9}Gd(CO)_6$ BP86

Gd	-0.48309000	0.11287200	0.00000000
C	-0.65460200	-1.99053200	1.36061300
C	-0.37094200	2.25772200	1.41910000
C	-0.37094200	2.25772200	-1.41910000
C	1.53179100	-0.40569800	1.27869100
C	-0.65460200	-1.99053200	-1.36061300
C	1.53179100	-0.40569800	-1.27869100
O	-0.75082700	-2.92330200	2.03268100
O	-0.15118300	3.13997700	2.12344500
O	2.45468700	-0.56428000	1.95749800
O	-0.75082700	-2.92330200	-2.03268100
O	-0.15118300	3.13997700	-2.12344500
O	2.45468700	-0.56428000	-1.95749800

$^{11}Gd(CO)_6$ BP86

Gd	0.00000000	0.00000000	0.00000000
C	0.00000000	0.00000000	2.48091000
C	0.00000000	2.48091000	0.00000000
C	2.48091000	0.00000000	0.00000000
C	-2.48091000	0.00000000	0.00000000
C	0.00000000	-2.48091000	0.00000000
C	0.00000000	0.00000000	-2.48091000
O	0.00000000	0.00000000	3.63469200
O	0.00000000	3.63469200	0.00000000
O	-3.63469200	0.00000000	0.00000000
O	0.00000000	-3.63469200	0.00000000
O	3.63469200	0.00000000	0.00000000
O	0.00000000	0.00000000	-3.63469200

$^{9}Gd(CO)_6$ B3LYP

Gd	-0.41434800	0.11024500	0.00000000
C	-0.71864200	-1.98713500	1.40782500
C	-0.40709600	2.25259700	1.47968500
C	-0.40709600	2.25259700	-1.47968500
C	1.61440400	-0.41932600	1.34376500
C	-0.71864200	-1.98713500	-1.40782500
C	1.61440400	-0.41932600	-1.34376500
O	-0.91672500	-2.87350200	2.09588900
O	-0.29627500	3.10936800	2.21721000
O	2.50389300	-0.56144700	2.04550100
O	-0.91672500	-2.87350200	-2.09588900
O	-0.29627500	3.10936800	-2.21721000
O	2.50389300	-0.56144700	-2.04550100

$^{11}Gd(CO)_6$ B3LYP

Gd	0.00000000	0.00000000	0.00000000
C	0.00000000	0.00000000	2.49796600
C	0.00000000	2.49796600	0.00000000
C	2.49796600	0.00000000	0.00000000
C	-2.49796600	0.00000000	0.00000000
C	0.00000000	-2.49796600	0.00000000
C	0.00000000	0.00000000	-2.49796600
O	0.00000000	0.00000000	3.63732300
O	0.00000000	3.63732300	0.00000000
O	-3.63732300	0.00000000	0.00000000
O	0.00000000	-3.63732300	0.00000000
O	3.63732300	0.00000000	0.00000000
O	0.00000000	0.00000000	-3.63732300

$^{8}Tb(CO)_8$ B3LYP

Tb	-0.00000000	0.00000000	0.00000000
C	-1.48795569	1.48827719	1.42659489
C	1.48827719	1.48795569	-1.42659489
C	-1.48827719	1.48795569	-1.42659489
C	1.48795569	1.48827719	1.42659489
C	1.48827719	-1.48795569	-1.42659489
C	-1.48795569	-1.48827719	1.42659489
C	1.48795569	-1.48827719	1.42659489
C	-1.48827719	-1.48795569	-1.42659489
O	2.15443036	2.16291168	-2.05320536
O	-2.16291168	2.15443036	2.05320536
O	2.16291168	2.15443036	2.05320536
O	-2.15443036	2.16291168	-2.05320536
O	-2.16291168	-2.15443036	2.05320536
O	2.15443036	-2.16291168	-2.05320536
O	-2.15443036	-2.16291168	-2.05320536
O	2.16291168	-2.15443036	2.05320536

$^{10}Tb(CO)_8$ B3LYP

Tb	0.00000000	-0.00000000	0.00000000
C	-1.43205512	1.39751730	-1.52815948
C	1.39751730	1.43205512	1.52815948
C	1.43205512	1.39751730	-1.52815948
C	-1.39751730	1.43205512	1.52815948
C	1.39751730	-1.43205512	1.52815948
C	-1.43205512	-1.39751730	-1.52815948
C	-1.39751730	-1.43205512	1.52815948
C	1.43205512	-1.39751730	-1.52815948
O	2.13102826	1.94126896	2.23206060
O	-1.94126896	2.13102826	-2.23206060
O	-2.13102826	1.94126896	2.23206060
O	1.94126896	2.13102826	-2.23206060
O	-1.94126896	-2.13102826	-2.23206060
O	2.13102826	-1.94126896	2.23206060
O	1.94126896	-2.13102826	-2.23206060
O	-2.13102826	-1.94126896	2.23206060

^{7}Dy(CO)$_8$ B3LYP

Dy	0.00000000	-0.00242900	0.00912000
C	1.48931700	-1.46079300	-1.41088900
C	-1.46203400	1.46547200	-1.43484200
C	1.46198900	1.46550300	-1.43485600
C	-1.48929800	-1.46082400	-1.41087700
C	-1.46852900	1.46323300	1.43072300
C	1.49149100	-1.46573800	1.40065200
C	-1.49144800	-1.46577000	1.40066600
C	1.46851200	1.46326200	1.43071000
O	-2.13065300	2.12943000	-2.07009800
O	2.16535300	-2.12052900	-2.04286900
O	-2.16532500	-2.12057400	-2.04285400
O	2.13058900	2.12947600	-2.07011700
O	2.17172600	-2.12923400	2.02466700
O	-2.14030400	2.12872600	2.06145100
O	2.14027900	2.12876700	2.06143400
O	-2.17166500	-2.12928100	2.02468400

^{9}Dy(CO)$_8$ B3LYP

Dy	0.02209600	0.00163100	-0.00438000
C	-0.34784600	1.88770500	-1.65715800
C	-0.35432500	-2.20949200	-1.20639600
C	-2.37308400	-0.10861600	-0.94306600
C	1.62759300	-0.22497900	-1.91997900
C	1.70724600	-1.34860200	1.30305800
C	-1.03093200	1.72380900	1.56201500
C	1.81093500	1.49016000	0.97581900
C	-1.15188700	-1.21359100	1.90925900
O	-0.47053200	-3.24020300	-1.67590100
O	-0.46416300	2.78514800	-2.34842600
O	2.40671200	-0.31840800	-2.74677100
O	-3.46446200	-0.14594300	-1.26099400
O	-1.53405900	2.53455300	2.18203600
O	2.49498600	-1.98595400	1.82391300
O	-1.71395800	-1.80959500	2.69896200
O	2.64741200	2.16964900	1.34565300

^{6}Ho(CO)$_8$ B3LYP

Ho	0.00000000	0.00000000	0.00000000
C	0.00000000	2.00970000	1.47685800
C	-2.00970000	0.00000000	-1.47685800
C	-2.00970000	0.00000000	1.47685800
C	0.00000000	2.00970000	-1.47685800
C	0.00000000	-2.00970000	-1.47685800
C	2.00970000	0.00000000	1.47685800
C	2.00970000	0.00000000	-1.47685800
C	0.00000000	-2.00970000	1.47685800
O	-2.91685400	0.00000000	-2.16253100
O	0.00000000	2.91685400	2.16253100
O	0.00000000	2.91685400	-2.16253100
O	-2.91685400	0.00000000	2.16253100
O	2.91685400	0.00000000	2.16253100
O	0.00000000	-2.91685400	-2.16253100
O	0.00000000	-2.91685400	2.16253100
O	2.91685400	0.00000000	-2.16253100

^{8}Ho(CO)$_8$ B3LYP

Ho	-0.00110500	-0.00000200	-0.00000800
C	0.85636400	-1.86149900	-1.46274100
C	-0.85216700	-1.82372600	1.51164900
C	-1.86186900	-0.87835000	-1.44581900
C	1.86574900	-0.83748600	1.46518900
C	-1.84823100	0.87502300	1.46533400
C	1.84839500	0.84125600	-1.48444600
C	0.86839600	1.86303000	1.45368500
C	-0.87204300	1.82156200	-1.50251500
O	-1.25812100	-2.68388900	2.13851700
O	1.26352800	-2.73763500	-2.06619300
O	2.74526300	-1.23914900	2.06742000
O	-2.73928800	-1.29642300	-2.04003400
O	2.72049700	1.24491200	-2.09606300
O	-2.71998700	1.29170500	2.06875600
O	-1.28660900	2.68067200	-2.12519100
O	1.28052900	2.73996600	2.05259900

^{6}Ho(CO)$_7$ B3LYP

Ho	0.00509300	0.05844800	-0.01063600
C	-0.04816400	2.53652700	-0.10665000
C	-1.99172800	0.16418300	-1.45201400
C	-2.00098900	0.35141800	1.38504100
C	0.01899600	-1.80510900	1.54298500
C	1.99467700	0.21177700	-1.45660600
C	2.00109900	0.39918200	1.38690200
C	0.01948100	-2.01692100	-1.27494800
O	0.02305200	-2.66990600	2.28549300
O	2.94170400	0.57457100	2.00334300
O	2.93284700	0.30141400	-2.09421500
O	-2.93411300	0.23030800	-2.08613700
O	-2.94767600	0.50208400	1.99853900
O	-0.07747900	3.67119400	-0.15201500
O	0.02398100	-2.97995900	-1.88446800

^{8}Ho(CO)$_7$ B3LYP

Ho	0.00000000	-0.00000000	0.04167596
O	0.00000000	-0.00000000	3.79723096
O	-3.14834706	-1.56688090	0.98455823
O	-2.17966816	1.35262744	-2.44137421
O	2.26124380	1.21133427	-2.44137421
O	2.93113219	-1.94310808	0.98455823
O	-0.08157565	-2.56396171	-2.44137421
O	0.21721487	3.50998898	0.98455823
C	-0.06228479	-1.81924341	-1.57194698
C	1.60665341	0.85568150	-1.57194698
C	2.00288584	-1.32037636	0.78485021
C	0.00000000	-0.00000000	2.66340896
C	-1.54436862	0.96356192	-1.57194698
C	-2.14492239	-1.07436184	0.78485021
C	0.14203655	2.39473820	0.78485021

$^{6}Ho(CO)_6$ B3LYP

Ho	0.00009600	0.00129600	0.30728200
C	1.56828400	-1.84175000	0.62343700
C	1.57792700	1.83264300	0.63205700
C	-1.57802500	1.83243900	0.63165700
C	1.41472900	0.00677500	-1.60843700
C	-1.56899400	-1.84100900	0.62316200
C	-1.41400500	0.00696800	-1.60880000
O	2.30739500	-2.70084700	0.74168300
O	2.32098100	2.68803000	0.75276800
O	2.11009100	0.00832600	-2.51533100
O	-2.30868000	-2.69962900	0.74128400
O	-2.32137500	2.68760000	0.75216500
O	-2.10915000	0.00862000	-2.51586300

$^{8}Ho(CO)_6$ B3LYP

Ho	0.00000000	0.00000000	0.00000000
C	0.00000000	2.02888663	1.37054815
C	1.75706736	1.01444331	-1.37054815
C	-0.00000000	-2.02888663	-1.37054815
C	1.75706736	-1.01444331	1.37054815
C	-1.75706736	1.01444331	-1.37054815
C	-1.75706736	-1.01444331	1.37054815
O	0.00000000	2.97282971	2.00773604
O	2.57454605	1.48641486	-2.00773604
O	2.57454605	-1.48641486	2.00773604
O	-2.57454605	1.48641486	-2.00773604
O	-0.00000000	-2.97282971	-2.00773604
O	-2.57454605	-1.48641486	2.00773604

$^{5}Er(CO)_8$ B3LYP

Er	-0.00000000	0.00000000	-0.00000000
C	0.00000000	1.99614666	1.46962650
C	0.00000000	-1.99614666	1.46962650
C	1.99614666	0.00000000	1.46962650
C	-1.99614666	-0.00000000	1.46962650
C	-0.00000000	-1.99614666	-1.46962650
C	-0.00000000	1.99614666	-1.46962650
C	-1.99614666	0.00000000	-1.46962650
C	1.99614666	-0.00000000	-1.46962650
O	0.00000000	-2.90000417	2.16039178
O	-0.00000000	2.90000417	2.16039178
O	-2.90000417	-0.00000000	2.16039178
O	2.90000417	0.00000000	2.16039178
O	0.00000000	2.90000417	-2.16039178
O	-0.00000000	-2.90000417	-2.16039178
O	2.90000417	-0.00000000	-2.16039178
O	-2.90000417	0.00000000	-2.16039178

$^{7}Er(CO)_8$ B3LYP

Er	0.00886400	-0.01598000	-0.00054300
C	-0.29892000	2.01104700	1.43594900
C	0.48007000	-1.98078800	1.46776600
C	2.08372400	0.41889900	1.32863400
C	-1.90276000	-0.35870300	1.57520500
C	1.59635800	-1.15422900	-1.55393700
C	-1.76395700	1.12528400	-1.35261100
C	-1.23863300	-1.70482600	-1.36410900
C	1.03774600	1.66062400	-1.53886300
O	0.70882200	-2.90986100	2.08628600
O	-0.47066700	2.96555700	2.03466100
O	-2.81253900	-0.52563000	2.24085000
O	3.06016200	0.64246100	1.87189800
O	-2.59524200	1.66837300	-1.91190800
O	2.35436100	-1.70350700	-2.20355600
O	1.52751900	2.46871200	-2.17562600
O	-1.84298200	-2.48325500	-1.93651900

$^{4}Tm(CO)_8$ B3LYP

Tm	0.00000000	0.00000000	0.00000000
C	0.00000000	1.98934876	-1.45457400
C	-1.98934876	0.00000000	1.45457400
C	0.00000000	1.98934876	1.45457400
C	-1.98934876	-0.00000000	-1.45457400
C	-0.00000000	-1.98934876	1.45457400
C	1.98934876	0.00000000	-1.45457400
C	0.00000000	-1.98934876	-1.45457400
C	1.98934876	-0.00000000	1.45457400
O	-2.90100385	0.00000000	2.13470500
O	0.00000000	2.90100385	-2.13470500
O	-2.90100385	-0.00000000	-2.13470500
O	0.00000000	2.90100385	2.13470500
O	2.90100385	0.00000000	-2.13470500
O	0.00000000	-2.90100385	2.13470500
O	2.90100385	0.00000000	2.13470500
O	-0.00000000	-2.90100385	-2.13470500

$^{6}Tm(CO)_8$ B3LYP

Tm	0.00111200	-0.00206600	0.00048200
C	-1.23371200	1.60296600	1.44087400
C	1.19713200	-1.61356900	1.45803700
C	1.59750100	1.20411200	1.47556500
C	-1.62084400	-1.21907500	1.43244200
C	2.02196700	-0.11266000	-1.43519900
C	-1.99792200	0.12541900	-1.47069600
C	-0.10634700	-1.99812100	-1.46693100
C	0.13774400	2.02210600	-1.43344700
O	1.76449800	-2.38616700	2.07451100
O	-1.82162300	2.37047000	2.04398700
O	-2.39339700	-1.79830500	2.03778800
O	2.36403500	1.77395500	2.09683600
O	-2.95256300	0.17856100	-2.09049500
O	2.98449100	-0.16532200	-2.04317700
O	0.20065900	2.98798700	-2.03476100
O	-0.15232900	-2.95174400	-2.08933300

^{4}Tm(CO)$_7$ B3LYP

Tm	-0.00003800	0.04041600	-0.00034100
C	-0.00076500	2.48108700	-0.00492700
C	-1.95414800	0.29305800	-1.42070700
C	-1.96988700	0.29736200	1.40227900
C	-0.00672500	-1.89333200	1.42757200
C	1.96911500	0.28988000	-1.40557800
C	1.95462600	0.30273400	1.41723200
C	0.00785500	-1.90321500	-1.41499300
O	-0.01058200	-2.79733200	2.12179100
O	2.88799400	0.44839200	2.05268200
O	2.90935400	0.42437000	-2.03276600
O	-2.88724700	0.43422100	-2.05753600
O	-2.91047300	0.43529500	2.02822700
O	-0.00108600	3.61767500	-0.00704200
O	0.01231300	-2.81189300	-2.10306900

^{6}Tm(CO)$_7$ B3LYP

Tm	0.04869798	-0.00019409	0.00000000
C	-1.54402659	1.79523140	0.00000000
C	-1.55422972	-0.85939316	1.58062668
C	0.81376063	1.20143798	2.01954376
C	2.60411501	-0.11173228	0.00000000
C	-1.55422972	-0.85939316	-1.58062668
C	0.81376063	1.20143798	-2.01954376
C	0.66618274	-2.40561190	0.00000000
O	3.73849002	-0.15119092	0.00000000
O	1.05079236	1.78555953	-2.96457077
O	-2.42898468	-1.20620290	-2.23116899
O	-2.42898468	-1.20620290	2.23116899
O	1.05079236	1.78555953	2.96457077
O	-2.40401083	2.55192813	0.00000000
O	0.81643209	-3.53126285	0.00000000

^{4}Tm(CO)$_6$ B3LYP

Tm	-0.26358793	0.01247094	0.00000000
C	-0.67267527	1.53412901	1.82257540
C	-0.67267527	1.53412901	-1.82257540
C	-0.63428659	-1.57437596	-1.76531718
C	1.61300327	1.45252267	0.00000000
C	-0.63428659	-1.57437596	1.76531718
C	1.63761987	-1.38738233	0.00000000
O	-0.84144195	2.25122600	2.69209165
O	-0.84144195	2.25122600	-2.69209165
O	2.50987937	2.16038955	0.00000000
O	-0.78954163	-2.33782411	2.59669671
O	-0.78954163	-2.33782411	-2.59669671
O	2.54149877	-2.08643646	0.00000000

^{6}Tm(CO)$_6$ B3LYP

Tm	0.00000000	0.00000000	0.00000000
C	0.00000000	2.41193500	0.00000000
C	0.00000000	0.00000000	2.41091200
C	2.41674400	-0.05376000	0.00000000
C	-2.41674400	0.05376000	0.00000000
C	0.00000000	0.00000000	-2.41091200
C	0.00000000	-2.41193500	0.00000000
O	-0.00304600	3.55136400	0.00000000
O	0.00000000	0.00000000	3.55033000
O	-3.55570500	0.07881700	0.00000000
O	0.00000000	0.00000000	-3.55033000
O	3.55570500	-0.07881700	0.00000000
O	0.00304600	-3.55136400	0.00000000

^{1}Yb(CO)$_8$ BP86

Yb	0.00000000	0.00000000	0.00000000
C	0.00000000	1.97349000	1.40960000
C	0.00000000	-1.97349000	1.40960000
C	1.97349000	0.00000000	1.40960000
C	-1.97349000	0.00000000	1.40960000
C	1.39546800	-1.39546800	-1.40960000
C	-1.39546800	1.39546800	-1.40960000
C	-1.39546800	-1.39546800	-1.40960000
C	1.39546800	1.39546800	-1.40960000
O	0.00000000	-2.89128900	2.10250200
O	0.00000000	2.89128900	2.10250200
O	-2.89128900	0.00000000	2.10250200
O	2.89128900	0.00000000	2.10250200
O	-2.04445000	2.04445000	-2.10250200
O	2.04445000	-2.04445000	-2.10250200
O	2.04445000	2.04445000	-2.10250200
O	-2.04445000	-2.04445000	-2.10250200

^{3}Yb(CO)$_8$ BP86

Yb	0.00000000	0.00000000	0.00000000
C	-0.40344600	2.00525500	1.39879500
C	0.40344600	-2.00525500	1.39879500
C	2.00525500	0.40344600	1.39879500
C	-2.00525500	-0.40344600	1.39879500
C	2.00525500	-0.40344600	-1.39879500
C	-2.00525500	0.40344600	-1.39879500
C	-0.40344600	-2.00525500	-1.39879500
C	0.40344600	2.00525500	-1.39879500
O	0.64670600	-2.93125400	2.03611900
O	-0.64670600	2.93125400	2.03611900
O	-2.93125400	-0.64670600	2.03611900
O	2.93125400	0.64670600	2.03611900
O	-2.93125400	0.64670600	-2.03611900
O	2.93125400	-0.64670600	-2.03611900
O	0.64670600	2.93125400	-2.03611900
O	-0.64670600	-2.93125400	-2.03611900

^{1}Yb(CO)$_8$ B3LYP

Yb	0.00000000	0.00000000	0.00000000
C	0.00000000	2.04778600	1.48289800
C	0.00000000	-2.04778600	1.48289800
C	2.04778600	0.00000000	1.48289800
C	-2.04778600	0.00000000	1.48289800
C	1.44800300	-1.44800300	-1.48289800
C	-1.44800300	1.44800300	-1.48289800
C	-1.44800300	-1.44800300	-1.48289800
C	1.44800300	1.44800300	-1.48289800
O	0.00000000	-2.94958200	2.17325200
O	0.00000000	2.94958200	2.17325200
O	-2.94958200	0.00000000	2.17325200
O	2.94958200	0.00000000	2.17325200
O	-2.08567000	2.08567000	-2.17325200
O	2.08567000	-2.08567000	-2.17325200
O	2.08567000	2.08567000	-2.17325200
O	-2.08567000	-2.08567000	-2.17325200

$^{3}Yb(CO)_8$ B3LYP

Yb	0.00000000	0.00000000	0.00000000
C	-1.49163059	1.49163059	1.49163059
C	1.49163059	-1.49163059	1.49163059
C	1.49163059	1.49163059	1.49163059
C	-1.49163059	-1.49163059	1.49163059
C	1.49163059	1.49163059	-1.49163059
C	-1.49163059	-1.49163059	-1.49163059
C	1.49163059	-1.49163059	-1.49163059
C	-1.49163059	1.49163059	-1.49163059
O	2.14744811	-2.14744811	2.14744811
O	-2.14744811	2.14744811	2.14744811
O	-2.14744811	-2.14744811	2.14744811
O	2.14744811	2.14744811	2.14744811
O	-2.14744811	-2.14744811	-2.14744811
O	2.14744811	2.14744811	-2.14744811
O	-2.14744811	2.14744811	-2.14744811
O	2.14744811	-2.14744811	-2.14744811

$^{1}Yb(CO)_7$ BP86

Yb	0.05545800	0.02998200	0.00000000
C	-2.38797600	-0.08045700	0.00000000
C	-0.68892400	1.13415800	-2.03267900
C	0.02840900	-1.83380000	-1.57589600
C	2.37821400	-0.72048200	0.00000000
C	-0.68892400	1.13415800	2.03267900
C	0.02840900	-1.83380000	1.57589600
C	1.21416900	2.15801700	0.00000000
O	3.45777000	-1.12071300	0.00000000
O	0.02840900	-2.71486800	2.31451300
O	-1.06973200	1.63865500	2.99569900
O	-1.06973200	1.63865500	-2.99569900
O	0.02840900	-2.71486800	-2.31451300
O	-3.53719100	-0.12753300	0.00000000
O	1.76427300	3.16998000	0.00000000

$^{3}Yb(CO)_7$ BP86

Yb	0.00000000	0.00000000	-0.05061300
C	-1.65572000	0.95593000	1.47159100
C	1.65572000	0.95593000	1.47159100
C	0.00000000	2.36593400	-0.61562000
C	0.00000000	0.00000000	-2.47314900
C	0.00000000	-1.91186100	1.47159100
C	-2.04895900	-1.18296700	-0.61562000
C	2.04895900	-1.18296700	-0.61562000
O	0.00000000	0.00000000	-3.62486500
O	-3.02594400	-1.74703000	-0.84764000
O	0.00000000	-2.81736800	2.17985800
O	2.43991200	1.40868400	2.17985800
O	0.00000000	3.49405900	-0.84764000
O	-2.43991200	1.40868400	2.17985800
O	3.02594400	-1.74703000	-0.84764000

$^{1}Yb(CO)_7$ B3LYP

Yb	0.01896700	0.00280300	0.00000000
C	-2.51177800	0.05846000	0.00000000
C	-0.63514900	1.18668100	-2.12051500
C	-0.00022900	-1.88105400	-1.68753000
C	2.40451000	-0.78875100	0.00000000
C	-0.63514900	1.18668100	2.12051500
C	-0.00022900	-1.88105400	1.68753000
C	1.33740800	2.13183700	0.00000000
O	3.47438900	-1.17609800	0.00000000
O	-0.00022900	-2.72357800	2.44891700
O	-0.95326200	1.70541900	3.08180500
O	-0.95326200	1.70541900	-3.08180500
O	-0.00022900	-2.72357800	-2.44891700
O	-3.64745700	0.08404500	0.00000000
O	1.94455200	3.09424800	0.00000000

$^{3}Yb(CO)_7$ B3LYP

Yb	0.00000000	0.00000000	-0.01382400
C	-1.75983400	1.01604100	1.49304100
C	1.75983400	1.01604100	1.49304100
C	0.00000000	2.43056400	-0.64952000
C	0.00000000	0.00000000	-2.51685400
C	0.00000000	-2.03208100	1.49304100
C	-2.10493100	-1.21528200	-0.64952000
C	2.10493100	-1.21528200	-0.64952000
O	0.00000000	0.00000000	-3.65473600
O	-3.06456200	-1.76932600	-0.90790300
O	0.00000000	-2.94901600	2.16304200
O	2.55392300	1.47450800	2.16304200
O	0.00000000	3.53865200	-0.90790300
O	-2.55392300	1.47450800	2.16304200
O	3.06456200	-1.76932600	-0.90790300

$^{1}Yb(CO)_6$ BP86

Yb	0.00000000	0.00000000	0.00000000
C	0.00000000	1.74399500	1.67271200
C	-1.51034400	-0.87199700	1.67271200
C	-1.51034400	-0.87199700	-1.67271200
C	1.51034400	-0.87199700	1.67271200
C	0.00000000	1.74399500	-1.67271200
C	1.51034400	-0.87199700	-1.67271200
O	0.00000000	2.52744100	2.51831200
O	-2.18882800	-1.26372100	2.51831200
O	2.18882800	-1.26372100	2.51831200
O	0.00000000	2.52744100	-2.51831200
O	-2.18882800	-1.26372100	-2.51831200
O	2.18882800	-1.26372100	-2.51831200

$^{3}Yb(CO)_6$ BP86

Yb	0.00000000	0.00000000	0.30109900
C	0.00000000	2.34904800	0.94960700
C	2.03433600	-1.17452400	0.94960700
C	1.67055400	0.96449500	-1.16683900
C	-2.03433600	-1.17452400	0.94960700
C	-1.67055400	0.96449500	-1.16683900
C	0.00000000	-1.92898900	-1.16683900
O	0.00000000	3.48912700	1.12570100
O	3.02167200	-1.74456300	1.12570100
O	-3.02167200	-1.74456300	1.12570100
O	-2.47978800	1.43170600	-1.84098200
O	2.47978800	1.43170600	-1.84098200
O	0.00000000	-2.86341200	-1.84098200

$^{1}Yb(CO)_6$ B3LYP

Yb	0.00000000	0.00000000	0.00000000
C	0.00000000	1.79761200	1.72044700
C	-1.55677800	-0.89880600	1.72044700
C	-1.55677800	-0.89880600	-1.72044700
C	1.55677800	-0.89880600	1.72044700
C	0.00000000	1.79761200	-1.72044700
C	1.55677800	-0.89880600	-1.72044700
O	0.00000000	2.58548400	2.54189900
O	-2.23909500	-1.29274200	2.54189900
O	2.23909500	-1.29274200	2.54189900
O	0.00000000	2.58548400	-2.54189900
O	-2.23909500	-1.29274200	-2.54189900
O	2.23909500	-1.29274200	-2.54189900

$^{3}Yb(CO)_6$ B3LYP

Yb	0.00000000	0.00000000	0.00000000
C	0.00000000	2.23211900	1.14394100
C	1.93307200	-1.11606000	1.14394100
C	1.93307200	1.11606000	-1.14394100
C	-1.93307200	-1.11606000	1.14394100
C	-1.93307200	1.11606000	-1.14394100
C	0.00000000	-2.23211900	-1.14394100
O	0.00000000	3.27523600	1.59981300
O	2.83643800	-1.63761800	1.59981300
O	-2.83643800	-1.63761800	1.59981300
O	-2.83643800	1.63761800	-1.59981300
O	2.83643800	1.63761800	-1.59981300
O	0.00000000	-3.27523600	-1.59981300

$^{2}Lu(CO)_8$ B3LYP

Lu	0.00000000	0.00000000	0.00000000
C	-1.44454000	1.39967400	-1.39335400
C	1.44454000	-1.39967400	-1.39335400
C	-1.44454000	-1.39967400	-1.39335400
C	1.44454000	1.39967400	-1.39335400
C	1.44454000	-1.39967400	1.39335400
C	-1.44454000	1.39967400	1.39335400
C	1.44454000	1.39967400	1.39335400
C	-1.44454000	-1.39967400	1.39335400
O	2.12497900	-2.04658000	-2.03559300
O	-2.12497900	2.04658000	-2.03559300
O	2.12497900	2.04658000	-2.03559300
O	-2.12497900	-2.04658000	-2.03559300
O	-2.12497900	2.04658000	2.03559300
O	2.12497900	-2.04658000	2.03559300
O	-2.12497900	-2.04658000	2.03559300
O	2.12497900	2.04658000	2.03559300

$^{4}Lu(CO)_8$ B3LYP

Lu	0.00000000	0.00000000	0.00000000
C	0.00000000	1.99409300	1.43104000
C	0.00000000	-1.99409300	1.43104000
C	1.99409300	0.00000000	1.43104000
C	-1.99409300	0.00000000	1.43104000
C	1.41003600	-1.41003600	-1.43104000
C	-1.41003600	1.41003600	-1.43104000
C	-1.41003600	-1.41003600	-1.43104000
C	1.41003600	1.41003600	-1.43104000
O	0.00000000	-2.95525400	2.04375400
O	0.00000000	2.95525400	2.04375400
O	-2.95525400	0.00000000	2.04375400
O	2.95525400	0.00000000	2.04375400
O	-2.08968000	2.08968000	-2.04375400
O	2.08968000	-2.08968000	-2.04375400
O	2.08968000	2.08968000	-2.04375400
O	-2.08968000	-2.08968000	-2.04375400

$^{2}Lu(CO)_7$ B3LYP

Lu	0.00000000	0.00000000	0.03153495
C	0.00000000	0.00000000	2.43831137
C	1.92421800	-1.41053899	0.30499700
C	1.92421800	1.41053899	0.30499700
C	0.00000000	1.39741834	-1.88588551
C	-1.92421800	-1.41053899	0.30499700
C	-1.92421800	1.41053899	0.30499700
C	0.00000000	-1.39741834	-1.88588551
O	0.00000000	2.08332943	-2.79667779
O	-2.85273900	2.05223808	0.45512581
O	-2.85273900	-2.05223808	0.45512581
O	2.85273900	-2.05223808	0.45512581
O	2.85273900	2.05223808	0.45512581
O	0.00000000	0.00000000	3.57474817
O	0.00000000	-2.08332943	-2.79667779

$^{4}Lu(CO)_7$ B3LYP

Lu	0.00000000	0.00000000	0.06180700
C	1.51789200	0.87635500	-1.54195200
C	-1.51789200	0.87635500	-1.54195200
C	0.00000000	2.34833400	0.74868300
C	0.00000000	0.00000000	2.58691300
C	0.00000000	-1.75271100	-1.54195200
C	2.03371700	-1.17416700	0.74868300
C	-2.03371700	-1.17416700	0.74868300
O	0.00000000	0.00000000	3.72217600
O	3.00238400	-1.73342700	0.94697900
O	0.00000000	-2.48467600	-2.42232500
O	-2.15179200	1.24233800	-2.42232500
O	0.00000000	3.46685500	0.94697900
O	2.15179200	1.24233800	-2.42232500
O	-3.00238400	-1.73342700	0.94697900

[2]Lu(CO)$_6$ B3LYP

Lu	0.00000000	0.00000000	0.21426800
C	1.72557800	1.56293400	0.68602200
C	-1.72557800	1.56293400	0.68602200
C	-1.72557800	-1.56293400	0.68602200
C	0.00000000	1.42030500	-1.63823100
C	1.72557800	-1.56293400	0.68602200
C	0.00000000	-1.42030500	-1.63823100
O	2.55391900	2.31812100	0.89229800
O	-2.55391900	2.31812100	0.89229800
O	0.00000000	2.12801800	-2.53577100
O	2.55391900	-2.31812100	0.89229800
O	-2.55391900	-2.31812100	0.89229800
O	0.00000000	-2.12801800	-2.53577100

[4]Lu(CO)$_6$ B3LYP

Lu	0.00000000	0.00000000	0.00000000
C	0.00000000	0.00000000	2.38184300
C	0.00000000	2.38184300	0.00000000
C	-2.38184300	0.00000000	0.00000000
C	2.38184300	0.00000000	0.00000000
C	0.00000000	-2.38184300	0.00000000
C	0.00000000	0.00000000	-2.38184300
O	0.00000000	0.00000000	3.52131600
O	0.00000000	3.52131600	0.00000000
O	3.52131600	0.00000000	0.00000000
O	0.00000000	-3.52131600	0.00000000
O	-3.52131600	0.00000000	0.00000000
O	0.00000000	0.00000000	-3.52131600
